# Enhancing cancer cell adhesion with clay nanoparticles for countering metastasis

**DOI:** 10.1038/s41598-019-42498-y

**Published:** 2019-04-11

**Authors:** Sahel N. Abduljauwad, Habib-ur-Rehman Ahmed

**Affiliations:** Civil & Environmental Engineering Department, King Fahd University of Petroleum & Minerals (KFUPM), Dhahran, Saudi Arabia

## Abstract

Cancer metastasis results from the suppression of adhesion between cancer cells and the extracellular matrix, causing their migration from the primary tumor location and the subsequent formation of tumors in distant organs. This study demonstrates the potential use of nano-sized clay mineral particles to modulate adhesions between tumor cells and with the surrounding extracellular matrix. Atomic force microscopy studies of live cell cultures reveal a significant increase in adhesion between tumor cells and their environment after treatment with different types of electrically charged clay nanoparticles. The enhancement of adhesion among cancer cells was further confirmed through scratch type of wound healing assay studies. To provide insight into the adhesion mechanisms introduced by the clay nanoparticles, we performed a molecular-level computer simulation of cell adhesions in the presence and absence of the nanoparticles. Strong van der Waals and electrostatic attractions modelled in the molecular simulations result in an increase in the cohesive energy density of these environments when treated with clay crystallites. The increase in the cohesive energy density after the sorption of clay crystallites on cell-cell and cell-extracellular matrix complexes lends weight to our strategy of using clay nanoparticles for the restoration of adhesion among cancer cells and prevention of metastasis.

## Introduction

Cell-cell and cell-extracellular matrix (ECM) adhesions play a fundamental role in governing the structural integrity of healthy tissue and in regulating cellular morphology, migration, proliferation, survival, and differentiation^1,2^. Cell-cell adhesion is mediated by molecules of the cadherin family, while cell-ECM adhesion is promoted through receptors including syndecans, dystroglycans, and integrins^3^. The down-regulation of these molecular systems, particularly those involving E-cadherins and integrins, is a key feature of cancer metastasis, whereby cancer cells detach from each other and from the ECM and migrate to other parts of the body via the lymphatic system or the blood stream^4^. In addition to down-regulation of E-cadherin, another molecule known as N-cadherin shows increased levels in migrating cancer cells, as this molecule helps the cancer cell to slip through blood vessels during migration.

During metastasis, adhesion-molecule-mediated cell forces, termed as specific adhesion, become suppressed, and leading to the release of cancer cells into the lymphatic system or the blood stream. Subsequently, upon invading other tissues and organs, adhesive function may be recovered, leading to theformation of new tumor colonies^5–9^. There are three general characteristics of cancer cells that make them distinct electrically from normal cells. High negative charges, loss of specific adhesion, and gain of non-specific adhesion are three typical characteristics of cancer cells. Several studies on cancer cell surface charges^10–13^ have shown that excessive secretion of lactate ions and sialic acid lead to the removal of the positive ions from the cell surface to the intracellular space, leaving behind the negative charges on the cell surface. In another study^14^, it was concluded that cancer cells bear higher non-specific van der Waals and electrostatic forces and higher negative surface charges compared to normal cells. Among non-specific adhesion forces on cell surfaces, van der Waals forces are the most significant, while electrostatic forces are less significant and may be modified by the presence of the salts^15^. The increase in negative surface charges and non-specific adhesive forces on the malignant cells (i.e., mediated by Columbic interactions between electrically charged entities or by van der Waals forces) also facilitate re-adhesion to the surfaces of the distant organs during metastasis. Although significant improvements have been achieved in both the early diagnosis and treatment of the primary tumor, metastatic tumors still cause ninety percent of the deaths in cancer patients^5,16,17^. The development of practical approaches for controlling and hindering the progression of metastasis by keeping cancer cells localized to their primary sites thus remains a crucial challenge.

The restoration of adhesion between tumor cells and the surrounding ECM at their primary location using biochemical agents has been proposed as an approach for controlling tumor cell migration and hence the successful retardation of the formation of metastatic tumors. However, attempts in this direction have failed to provide significant and practical solutions. The use of heparins to retard metastasis via their anticlotting properties and their interactions with selectins and integrins have remained inconclusive^18^. Another study^19^ demonstrated the targeting by liposome nanoparticles of triple-negative murine breast-cancer metastasis by post-intravenous administration, but their ability to prevent the onset of metastasis, perhaps by targeting the pre-metastatic niche, is still uncertain. Several *in vitro*, *in vivo*, and epidemiological studies have hypothesized that epigallocatechin-3-gallate (the major component found in green tea) may inhibit tumor invasion and angiogenesis, which are essential mechanisms of tumor growth and metastasis^20^. However, this approach, too, has been inconclusive.

**Figure 1:**
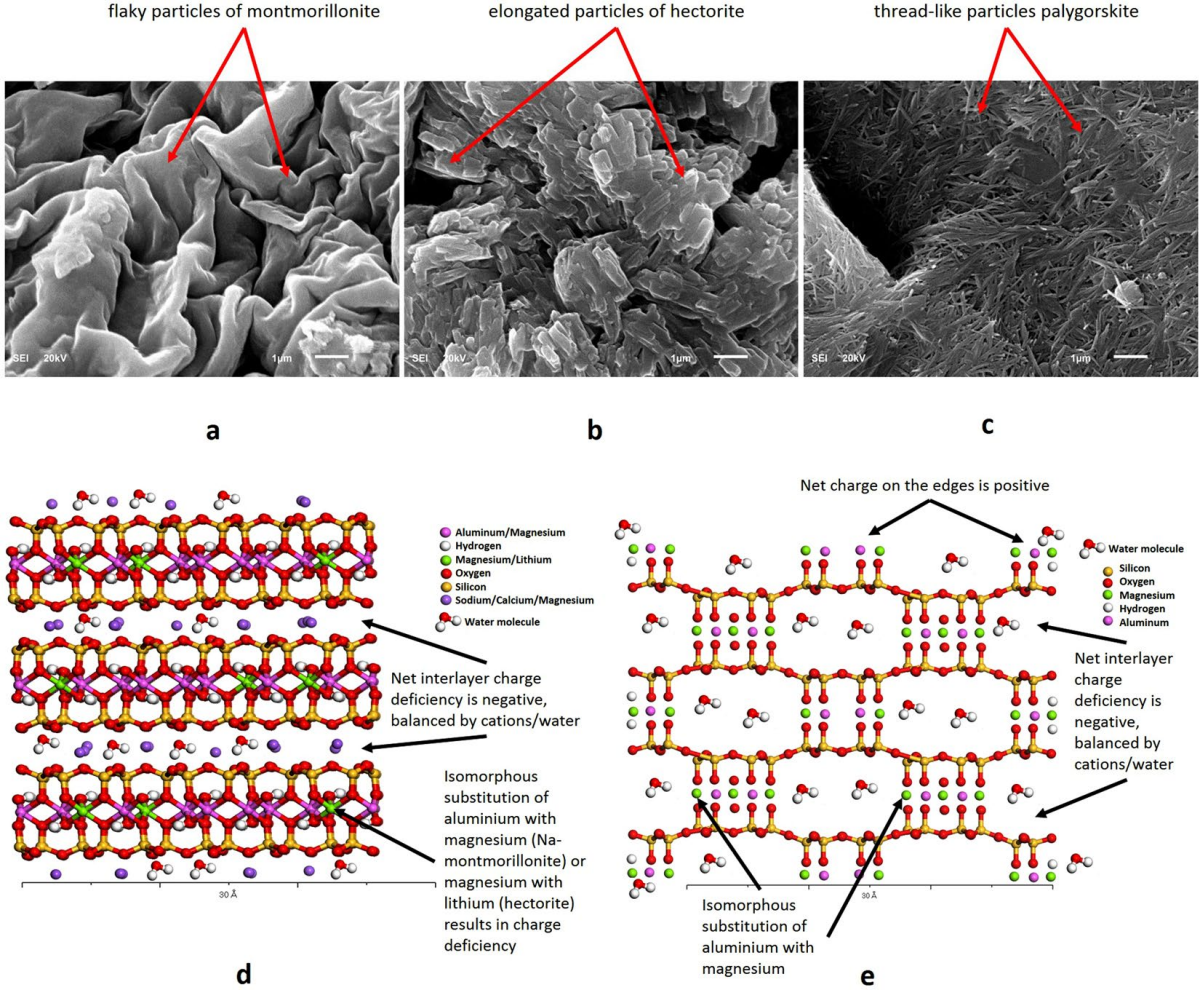
SEM images showing the configuration of different types of clays, (**a**) montmorillonite, (**b**) hectorite, and (**c**) palygorskite, and the corresponding molecular structure of the clays showing isomorphous substitution, charge deficiency, and interlayer cations, (**d**) Na-montmorillonite/hectorite (**e**) palygorskite.

Considering high negative surface charges and non-specific adhesive forces of the cancer cells, an alternative biophysical approach for restoring adhesion, could be the use of inorganic nano-sized charged clay mineral particles. Clay minerals are layered or tubular-structured phyllosilicates (Fig. 1a–c) typically characterized by a highly charged and large surface areas. Owing to these characteristics, the clay minerals could be considered suitable candidates for the restoration of the adhesion among cancer cells. The present study involved *in vitro* experiments and computer simulations of clay nanoparticle interactions with cancer cells and the ECM protein. Raji cancer cells, a human lymphoma cell line^21,22^ were used for the *in vitro* studies. Raji cells, like any other type of cancer cells, are negatively charged and own high non-specific adhesive forces such as van der Waals. These characteristics of cancer cells make them suitable candidates to be adhered by the charged clay nanoparticles. The ECM protein fibronectin (FN) was used to simulate the ECM and interact with the cells. FN is a major and multifunctional, extracellular matrix glycol protein composed of two nearly identical disulfide bound polypeptides of molecular weight 220 kDa. FN is positively charged protein that serves as cell adhesion molecule by anchoring cells to collagen and plays an important role in cell adhesion, growth, migration, and differentiation.

In this study, the adhesion measurements were performed in cell-cell, cell-ECM, and cell-cell-ECM configurations, both in the absence and presence of clay particles using atomic force microscopy (AFM). An experimental procedure has been developed at School of Medicine, University of Miami^23–25^ to measure the mechanical force and work required to detach two interacting cells using AFM. The technique is illustrated in Fig. S3. The overall strength of adhesion experienced by a given cell was quantified in terms of both (1) the maximum force detected by the AFM cantilever during cell-substrate separation, and (2) the total mechanical work done during de-adhesion, expressed by the shaded area in Fig. S3b. Schematics of cell-cell, cell-ECM, and cell-cell-ECM environments consisting of cancer cells and/or the ECM protein on the substrate and the cantilever attached with the cancer cell are shown in Fig. S3c. The purpose of the AFM experimentation was to find out the enhancement of the adhesion strength after the addition of the clay nanoparticles to the substrate components.

During the cell migration and invasion processes associated with cancer metastasis, there are diverse mechanisms that cells can employ to initiate and progress invasion. Scratch type of wound healing assays is one of the techniques employed for the evaluation of the clay particles as an anti-metastatic therapy. These assays are helpful in assessing the cell migration as the movement of individual cells, cell sheets and clusters from one location to another. Although scratch assays have inherent limitations of their inability to achieve reproducible and quantitative results, it is still an acceptable qualitative measurement tool to be used as complement to AFM studies. In this study, a confluent layer of breast cancer cells (MCF7) with and without three types of clay particles were scratched by a glass tube to create cell free area (schematically shown in Fig. S5). The configuration was imaged 24 hrs later to measure percent closure of the scratched area in the specimens with each type of clay.

Smectites and palygorskites, two main classes of clay minerals, display charge deficiency. This effect occurs mainly as a result of isomorphous substitution in their molecular structure and is balanced by cations and water molecules sorbed onto their surfaces (Fig. 1d,e). The clay nanoparticles are the main source of cohesion/adhesion in the soils and the argillaceous rocks. Considerable research by the authors have resulted in the detailed characterization and behavior evaluation of the charged clay minerals^26–28^. The individual clay nanoparticles join at the edges and ends to form several time bigger particles and then adhere, coat, and bridge the non-clay mineral particles present in soils to impart adhesion. In addition, the charged structure and large surface area of clay nanoparticles give them an affinity for charged entities, as found on bacterial surfaces and bacterial toxins. They have therefore been used as alternative medicine for several ailments^29–33^. Due to their adhesive nature, clay nanoparticles have also been used as sustained release medicine carriers^34,35^. The high affinity of the clay nanoparticles to charged surfaces, combined with the increased non-specific adhesion affinity of cancer cells, makes these charged clay particles potentially promising candidates to adhere to the cancer cells and hence control metastasis. Clay nanoparticles join with each other at their edges/ends to form bigger particles that would envelope and adhere to the multiple cancer cells and their environment mainly through electrostatic, van der Waals, and the zeta potential (ZP) interactions among individual cells and the clay nanoparticles. Clay nanoparticle flocks would also provide the bridging across the multiple cancer cells. The underlying idea of this novel approach is that, once bound to clay particles, cancer cells would be constrained from migrating to other parts of the body, thereby allowing more effective local therapeutic treatments.

We demonstrated the possible use of three charged clay minerals to restore adhesion between cancer cells and the ECM: Na-montmorillonite (SWy-3), hectorite (SHCa-l), and palygorskite (PFl-l). Scanning electron micrographs (SEM) and molecular structure of these clays are shown in Fig. 1a–c. The purpose of selecting three different clays was to study the relative effects of the surface charge intensity, surface area, cation exchange capacity (CEC), and the structural morphology/configuration. Each of the three electrically active clay minerals used in this study has its own distinct morphology, characteristics, and interaction behavior. To confirm the contribution to the adhesion by the charges on these clays, the experiments were also repeated using a neutral/uncharged kaolinite (KGa-1) clay. Based on the relatively higher adhesion enhancement of cell-cell configuration by Na-montmorillonite and cell-ECM by palygorskite, different proportions of a mixture of these two clays were also tested on the combined cell-cell-ECM assembly (Fig. S3). The physical, chemical, and mineralogical properties of the clay samples, obtained from the Clay Minerals Society (CMS)^36,37^, are summarized in Tables S1 to S3, while the details of the structures of these clays are provided in the following paragraphs.

Na-montmorillonite and hectorite are layered phyllosilicate clay smectites. In the colloid form, the space between adjacent layers can contain mobile sodium, calcium or magnesium cations that can become electrostatically attracted to external negatively charged surfaces^37^. Owing to the extent of the isomorphous substitution in the clay layers, Na-montmorillonite and hectorite have net negative charges of −0.55 and −1.57, respectively, on their flake-like interlayer surfaces (Table S1). The isomorphous substitution in hectorite results in the complete replacement of aluminum with magnesium and lithium in the octahedral sheet. Morphologically, Na-montmorillonite exists as equidimensional flakes or sheets with a size of approximately 0.5 × 0.5 × 0.001 microns in the dry powdered state (Fig. 1a and d). Hectorite particles, however, exist as elongated flakes of approximately 0.5 × 0.05 × 0.001 microns in their dry powdered state (Fig. 1b and d). These equidimensional/elongated flakes have negatively charged surfaces and positively charged edges and stalk over each other to form particle layers. The negative charges on their interlayer surfaces are balanced by the cations. In colloidal form, these cations dissociate from these clay particle surfaces and interact with the other negatively charged surfaces. The flaky particles of Na-montmorillonite exist mostly as equidimensional flakes, while those of hectorite are elongated and rectangular in dimension. These particles also have positively charged edges due to the presence of the broken bonds at their ends.

As contrast to smectites, Palygorskite has a lathe or thread-like structure with alternating regions of negative and positive charges that allow attraction to external surfaces of either charge. Palygorskite has a net interlayer charge of −2.07 in addition to the alternate positively and negatively charged edges along the entire outer surface of the thread-like particles (Table S1 and Fig. 1c and e). Morphologically, the palygorskite particles are tubular in shape. They also differ from the other layered silicates as they lack continuous octahedral sheets. Due to exposure of oxygen and hydrogen on the alternate edges, alternate negative and positive charges prevail along the outer surface of the tubular structure. This feature is unique to palygorskite and makes it versatile in being attracted to either positively or negatively charged surfaces. Since the octahedral sheet in palygorskite is discontinuous, some octahedral magnesium ions are exposed at the edges and hold bound water molecules (OH_2_). These magnesium ions are also available for electrostatically bonding to negatively charged surfaces when clay is present in the colloid form. In addition to the bound water, variable amounts of zeolitic (i.e., free) water (H_2_O) are contained in the rectangular channels.

In addition to the net surface charges, high surface area and the ZP also plays a central role in defining the interactions of these clay colloids with the cell-cell-ECM environment. The surface area of the nanoparticles is an essential measure of the extent of the interaction with the surrounding media. Palygorskite has the highest surface area of 136 m^2^/g, while Na-montmorillonite and hectorite have surface areas of 32 and 63 m^2^/g respectively. Higher surface areas promote larger bonding forces among the electrostatically oppositely charged surfaces and the van der Waals attractions. ZP defines the dispersion or flocculation tendency in the colloid form and the possible interactions with the other constituents in the suspensions or fluid media. Generally, a high zeta potential (>30 mV, either positive or negative) leads to dispersion, while a low zeta potential (<5 mV) can lead to agglomeration. High dispersion tendencies of the clay nanoparticles used in the study (ZP = −24 to −32 mV) result in the availability of high surface area maximizing the interactions with the cancer cells.

To gain insight into all the detailed mechanisms discussed above and considered underlying the promotion of adhesion by the clay nanoparticles, we also performed molecular-level simulations of their interaction with cells and the ECM. By modeling the essential components of integrin-mediated cellular binding in the presence and absence of representative clay nanoparticles, the simulations determine the contributions of electric charges in the nanoparticles to the cohesion of the model. Electrostatic and van der Waals Cohesive Energy Density (CED) concepts developed by the authors in the previous publications and patents^38–44^ were used in these simulations to compare the results in AFM measurements. Monte Carlo and molecular dynamics based procedures were used to simulate the interactions of clay nanoparticles with the cell components. Molecular simulations established and verified the relative levels of cohesiveness generated in cell-cell and cell-ECM system by different clay nanoparticles through electrostatic and van der Walls interactions. In addition, SEM was used to image the individual clays and the physical adherence of the different types of clays to various cell-cell-ECM culture environments. SEM results were used to explain the physical phenomena (coatings, enveloping, bridging etc.) responsible for the extent and degree of adhesion created among cancer cells by different clay nanoparticles.

## Results

### Nanoparticle morphology and size distributions

**Figure 2:**
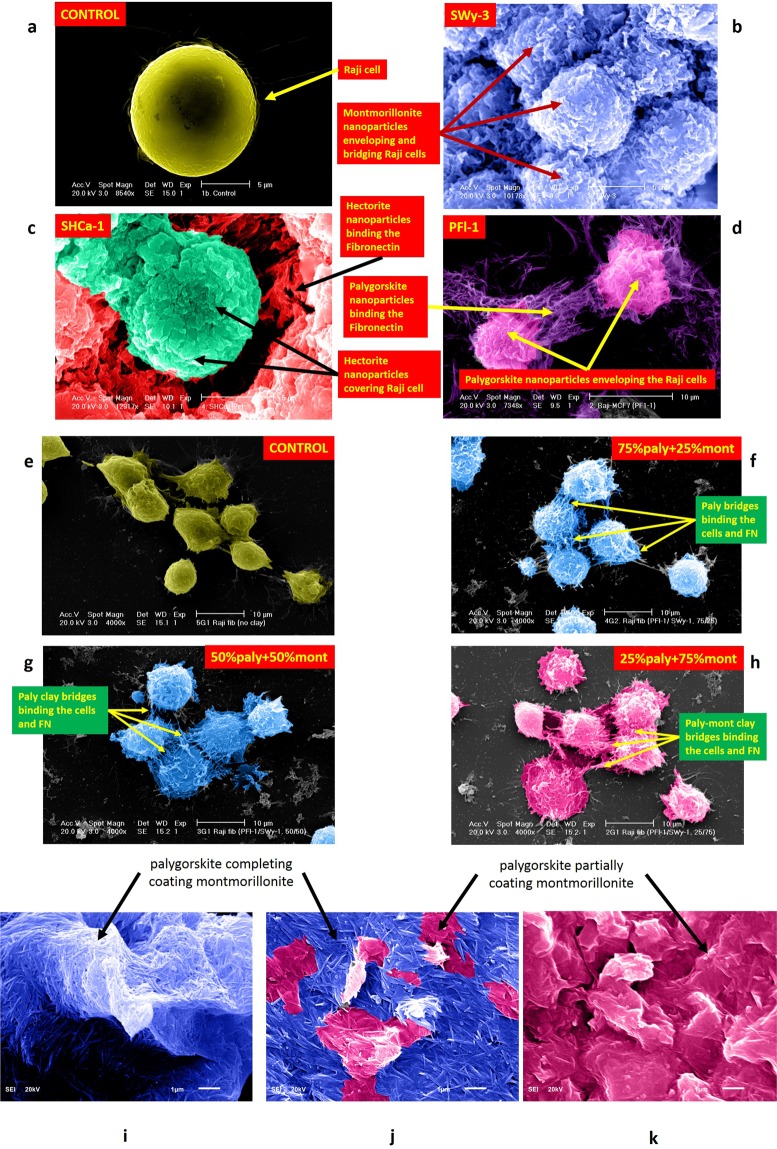
SEMs of the Raji-Raji-FN interactions with individual (**a**–**d**) and mixture (**e**–**h**) of clay nanoparticles. (**a**) Raji cell, (**b**) blue color represents a continuous coating of the Raji cells with Na-montmorillonite sheets, (**c**) green color represents small discrete hectorite particles covering the individual cell, while red color indicates hectorite particles embedded and binding the fibronectin, (**d**) pink color shows the palygorskite particles network covering the individual cells, while purple reveals the palygorskite particles web partially bridging the cells and partially binding the fibronectin with the cells. Clay mixtures (**e**) no clay (**f**) 75%paly + 25%mont, (**g**) 50%paly + 50%mont, and (**h**) 25%paly + 75%mont. SEMs of clay mixture (**i**) mont/play = 25/75: palygorskite particles could be seen completely covering the Na-montmorillonite particles, (**j**) mont/paly = 50/50: almost all the Na-montmorillonite particles are covered by palygorskite except some areas where the coating is lesser dense, (**k**) mont/paly = 75/25: palygorskite can be seen partially coating the Na-montmorillonite particles.

Purity of the clay specimens obtained after processing the natural samples from CMS through deflocculation, centrifuge, and sedimentation was confirmed using X-ray diffraction (XRD) analysis (Fig. S1). Particle size distribution plots for these pure clay samples, measured using Microtrac are shown in Fig. S2. Mean particle size (D_50_) for the Na-montmorillonite, hectorite, and palygorskite are 48, 51, and 27 nm respectively in deionized water, while these are 409, 248, and 221 nm respectively in RPMI. Maximum particle size assessed from D_90_ for these clays were about 1.5 to 2 times larger than the mean size. Due to zeta potential of −24 to −32 mV, the clay nanoparticles have a general tendency of dispersion in deionized water. However, owing to the relative flocculation tendency of clay nanoparticles in salt rich medium, it could be noted that the individual clay nanoparticles flocculate in RPMI medium to form larger particles. In RPMI, particle size increased about 8 times for Na-montmorillonite and palygorskite, while it is about 5 times for hectorite. It could be noted that although palygorskite particle size in RPMI is about 8 times than in the deionized water, it is still the smallest among the 3 clays. As observed in SEM (Fig. 2a–d), the clay particles further join at their edges and ends to form even bigger particles once get precipitated from the clay saturated RPMI medium during the interactions with Raji cells and FN. Based on the relative particle size of these clays, Na-montmorillonite forms largest particles among the three clays when precipitated from RPMI. This phenomenon is obvious from the SEMs in Fig. 2a–d, where flocculated Na-montmorillonite particles are large enough to cover and bridge the Raji cells of about 5 to 10 μm in diameter. It could be noted from Fig. 2b, that Na-montmorillonite particles completely cover the Raji cells due to large equidimensional flaky particles flocculated at the edges. On the other hand, hectorite and palygorskite, due to their relatively smaller elongated/thread-like particles, are not able to completely cover and overlap the cells (Fig. 2c and d). Hectorite and palygorskite could, however, be observed to be effectively binding the FN and the Raji cells through their thread-like particles (Fig. 2c and d). Na-montmorillonite particles, on the other hand, do not show any such anchorage type of binding of the cell with FN.

Despite relatively bigger particle sizes of the individual clays, mixtures of Na-montmorillonite and palygorskite have been observed to have much finer gradations (Fig. S2). The mixtures have finer gradations even in RPMI medium that would have resulted, otherwise, in bigger flocculated particles. Na-montmorillonite and palygorskite mixtures with 25/75, 50/50, and 75/25 proportions, prepared in RPMI medium, result in mean size of 25, 34, and 49 nm respectively. It could further be observed from Fig. S2, that higher the percentage of palygorskite in the mixture, finer is the resulting gradation. SEMs of palygorskite and Na-montmorillonite interacting with cell-cell-FN (Fig. 2e–h**)** and the high resolution focused images of the clays (Fig. 2i–k) also support the fact that there is a predominance of palygorskite thread-like particles in 50/50 and 25/75 Na-montmorillonite/palygorskite mixtures treated cells-FN, and all the Na-montmorillonite particles seem to be completely covered by the several layers of palygorskite particles. On the other hand, the resulting particles in the 75/25 proportioned mixture seem to have equal representation of both the clays. These phenomena have been further delineated in the SEMs of these clay mixtures in Fig. 2i–k. Fig. 2i and j represent the complete coating of the Na-montmorillonite by palygorskite, while the latter is partially and equally interacting with the former in Fig. 2k.

### Adhesion restoration of the cancer cell-cell, cell-ECM, and cell-cell-ECM configurations

**Figure 3:**
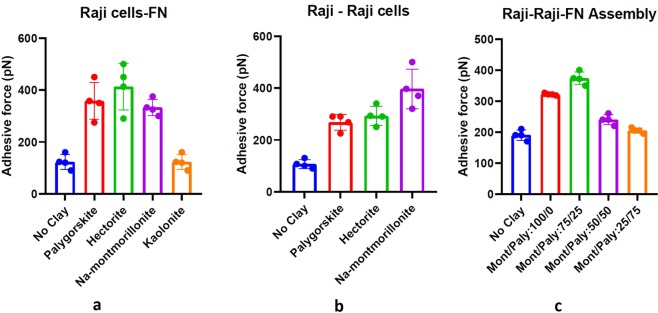
Summary of adhesion force measurements among (**a**) Raji cell to fibronectin (**b**) Raji cell to Raji cell and (**c**) Raji-Raji-FN assembly using AFM, before and after treatment with various proportions of Na-montmorillonite and palygorskite clay nanoparticles. Error bars represent the variations in adhesive force in three trials.

Figure 3 summarizes the effect of the three charged clay nanomaterials on adhesion properties. As evident from Fig. 3a and b, there is a significant increase in adhesive force among the Raji cancer cells and Raji cells and the fibronectin when any of these three clay particles were present at a saturated concentration of 0.2 mg/ml in RPMI. The adhesion test results for Raji-Raji cells, summarized in Fig. 3a, there is a general increase in adhesion among Raji cells, ranging from 175% (palygorskite and hectorite) to 285% (Na-montmorillonite). Similarly, as shown in Fig. 3b, there is a general increase in adhesion between Raji cells and fibronectin in the range of 160% (Na-montmorillonite), 195% (palygorskite)and 225% (hectorite).

Adhesion measurements of the mixtures of Na-montmorillonite and palygorskite in proportions of 25/75 and 50/50 reveal slight increase in adhesion (5 and 20% respectively) among Raji cells and FN configuration, while proportion of 75/25 has resulted in an increase in adhesion by 100% (Fig. 3c). Use of Na-montmorillonite alone without palygorskite on cell-cell-ECM configuration, however, resulted in an increase in adhesion by about 70%. So, maximum increase in adhesion in a Raji cells and FN environment is facilitated by Na-montmorillonite and palygorskite in a proportion of 75/25.

In contrast, as shown in Fig. 3b, the mean maximum adhesive force in the case of kaolinite (the neutral/uncharged clay mineral) varies significantly less relative to the baseline value (untreated samples) than the variation observed between the different trials, for both the cell-cell and the cell-ECM experiments.

**Figure 4:**
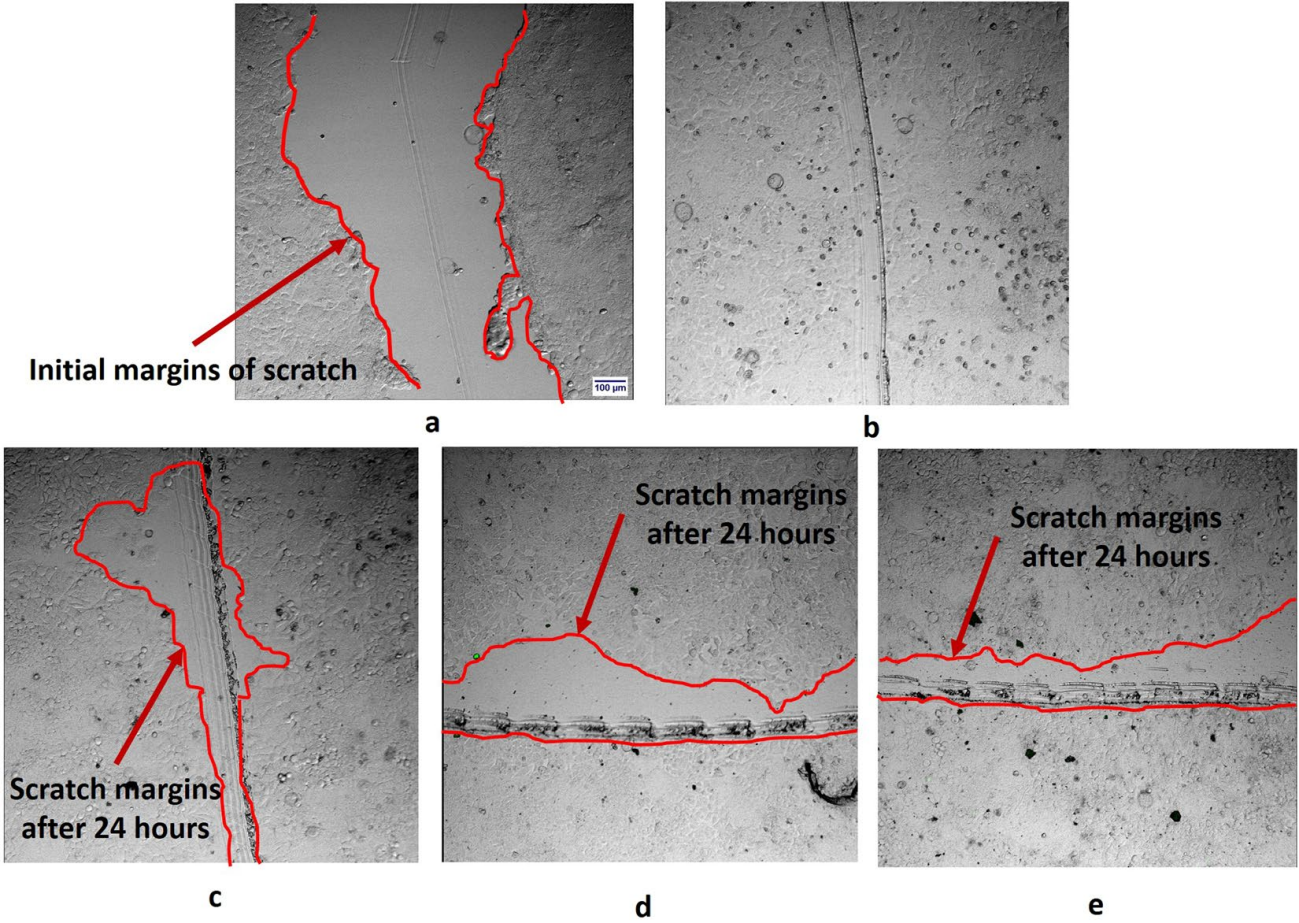
Microscopic views of scratch induced wound healing assays, (**a**) Scratch created in MCF7 cells without clay using glass tube: Cell migrations into the scratched area in the specimens after 24 hours (**b**) without clay, (**c**) with palygorskite (PFl-1), (**d**) with Na-montmorillonite (SWy-3), (**e**) with hectorite (SHCa-1).

Results of scratch-induced wound healing assays (Fig. 4) indicate that all the three clays have resulted in the reduction of the migration of MCF7 cells while there was a complete closure of the induced wound when no clay was used. Analysis of the images of the specimens with clay particles after 24 hours show a mean closure of the scratch by 59 + 3%, 42 + 4%, and 51 + 4% respectively for palygorskite, Na-montmorillonite, and hectorite.

### Molecular-level simulations

**Figure 5:**
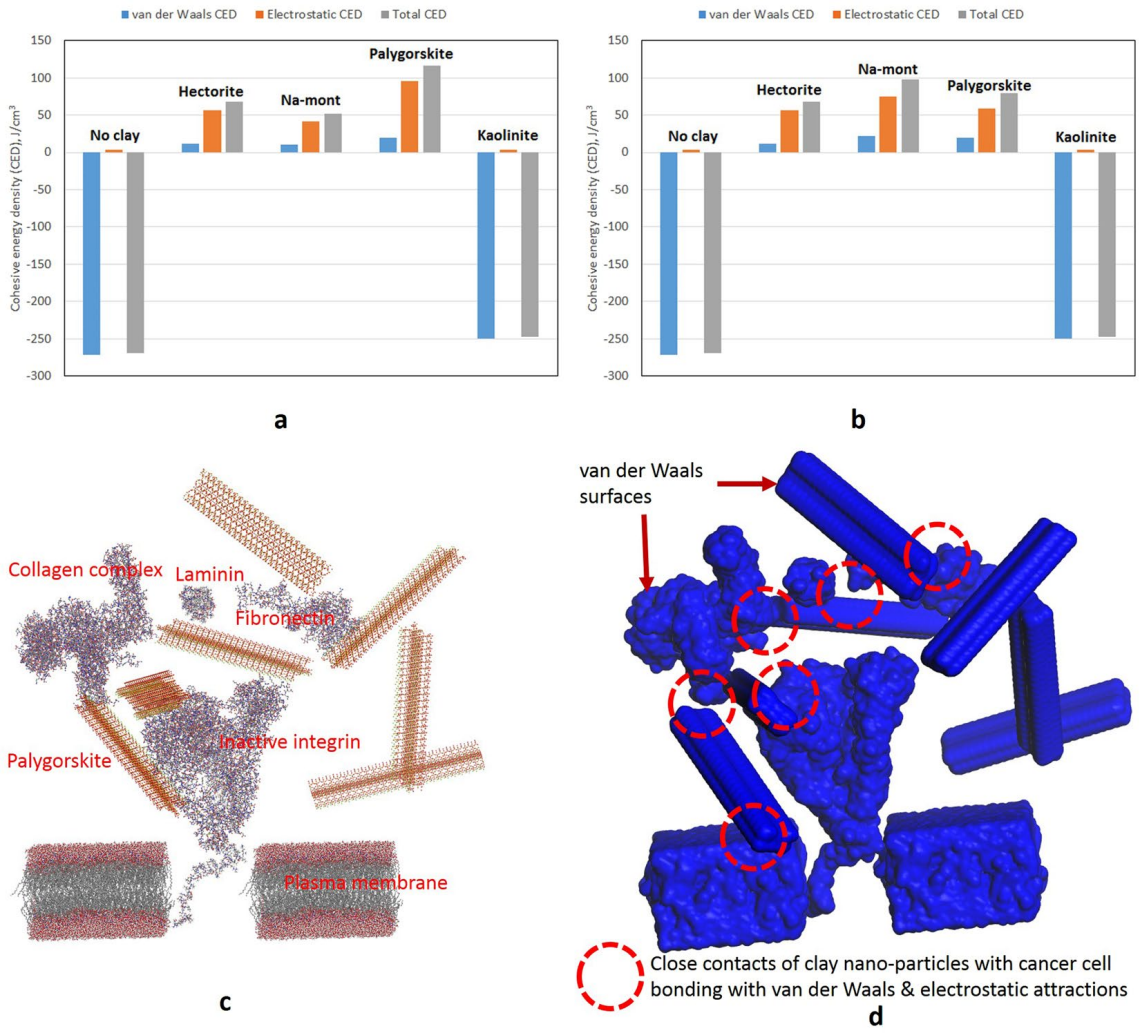
Summary of the electrostatic, van der Waals, and total cohesive energy density (CED) determined by the molecular-level simulations for (**a**) cell-ECM, (**b**) cell-cell configurations, without and with various types of clay nanoparticles. Equivalent molecular-level interactions of (**c**) seven crystallites of palygorskite and inactive integrin cell-ECM model, (**d**) van der Waals surfaces generated on clay, cell, and ECM components.

The Na-montmorillonite, hectorite, and palygorskite crystallites sorbed on the cell-ECM and cell-cell configurations in Fig. S8 show that the crystallites, due to their charged nature, generally orient themselves with respect to cell-ECM components/proteins. The specific orientations of the clay crystallites result from the charges on their surfaces and edges and on the cell-cell or cell-ECM configurations. Na-montmorillonite and hectorite bear negatively charged surfaces and positively charged edges and ends, while palygorskite has alternating positively and negatively charged zones along their tubular structure. Electrostatic and van der Waals cohesive energy densities of the cell-ECM and cell-cell interacting without and with various clay crystallites are plotted in Fig. 5. It could be noted that the electrostatic CED is almost absent in the cell-cell and cell-ECM configuration without clay nanoparticles. However, highly repulsive van der Waals CED exists among these configurations. Therefore, the cell-cell and cell-ECM configurations have an overall repulsive field typically representing a cancer cell environment. In Fig. 5a, it could be noted that higher attractive van der Waals CED by all the active clay crystallites reverses the repulsive van der Waals CED intrinsic in cell-cell and cell-ECM configurations. It could also be observed that palygorskite has contributed to a maximum increase in electrostatic attractive CED compared to its counterparts, i.e., Na-montmorillonite and hectorite. Similarly, as in case of the electrostatic CED, palygorskite has also contributed the highest van der Waals CED to the systems. Consequently, palygorskite results in the creation of the maximum total cohesive energy density and hence the highest adhesion among the cell-ECM configuration. Palygorskite, due to the presence of alternating negatively and positively charged surfaces along its tubular structure and higher van der Waals energies, has developed maximum adhesion to the cell-ECM structure. On the other hand, it could be noted in case of the cell-cell configuration (Fig. 5b), interaction with Na-montmorillonite has resulted in the highest electrostatic and van der Waals CED. Contribution of van der Waals attractions to overall CED of the clay and cell environment could be visualized from the high number of close contacts among van der Waals surfaces generated by the software in Fig. 5c.

## Discussion

**Figure 6:**
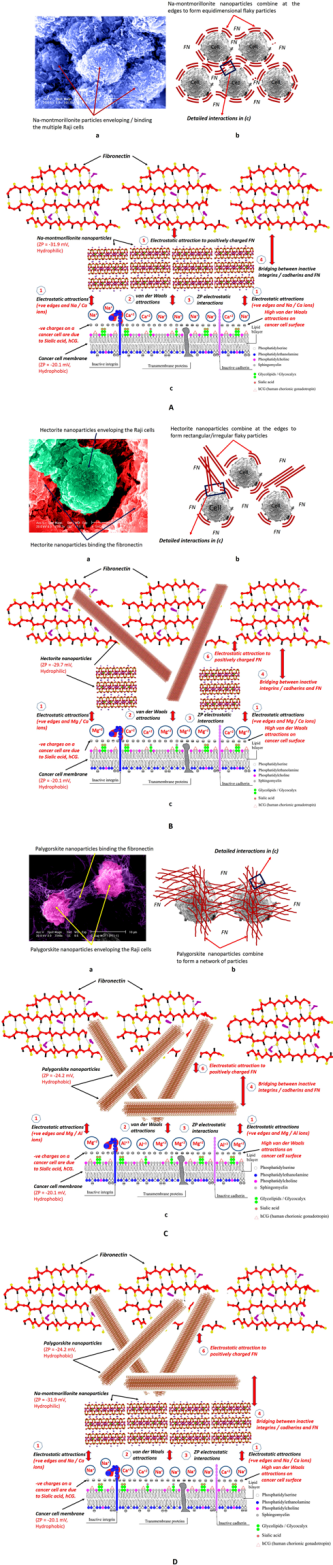
Interaction mechanisms developed between Raji cells and the surrounding fibronectin with various clay minerals (**A**) Montmorillonite (SWy-3), (**B**) Hectorite (SHCa-1), (**C**) Palygorskite (PFl-1), (**D**) montmorillonite-palygorskite mixed clays. In each of these interaction diagrams, (a) and (b) respectively are the SEM and the corresponding schematic sketch of the section of the Raji cells and the surrounding fibronectin with the respective clay particles binding and bridging the cells, (c) Several possible mechanisms of interactions of the respective clays with the cancer cells and their environment. (1) Electrostatic attraction between positively charged nanoparticle edges and Na/Ca/Mg ions with negatively charged cell surfaces, (2) Van der Waals attractions, (3) ZP electrostatic interactions, (4) Bridging between cells and the non-functional cadherins of adjacent cancer cells by the clay particles, (5) Electrostatic attractions and bridging between cells and the non-functional integrins and FN by the montmorillonite clay particles, (6) Electrostatic attraction of hectorite, palygorskite, and mixed (montmorillonite and palygorskite) clay particles’ edges and ends to the positively charges fibronectin and thus bridging over the cadherins.

Due to high negative charges and van der Waals forces on cancer cells and positive charges on FN, the charged clay particles have proven to have a natural tendency to get attracted to the cell-FN assemblies. Based on the characterization and behavior of the Raji cells, fibronectin, and the clay nanoparticles, observed in AFM results, particle size distribution, ZP, molecular-level simulations and visualizations from SEMs, three cell-cell-ECM-clays interaction models have been sketched. Interaction models for Na-montmorillonite, hectorite, and palygorskite are shown in Fig. 6A–C, while the one for the mixture of Na-montmorillonite and palygorskite is presented in Fig. 6D. Each model consists of an SEM in (a), a corresponding schematic cross-section of SEM in (b), and a comprehensive interaction model in (c). In each of the interaction models shown in Fig. 6A–D, several different modes of interactions have been identified and are marked on the corresponding sketches. These modes generally constitute attractions due to electrostatic and van der Waals forces, ZP electrostatic interactions, and bridging between cell-cell and cell-FN. Each mode contributes to the overall adhesiveness of the system to a different degree and extent depending on the clay type.

### Individual clay nanoparticles interaction models

Sodium, calcium, and magnesium cations held in the interlayers of the clay nanoparticles are dispersed in RPMI medium in the colloidal form and thus create another positively charged ion layer attaching to the negatively charged cell surfaces. This mechanism reduces the repulsion between negatively charged surfaces of these clays and the cell surfaces, bringing them closer and hence further promoting the bonding of the clay particles on cell surfaces through van der Waal forces and ZP interactions. These interaction mechanisms are marked as 1, 2, and 3 in Fig. 6A,B and C. In addition to electrostatic attractions, van der Waals interactions seem to be dominating in the adhesion process. Relatively higher van der Waals CED than the electrostatic ones (290 vs 70 J/cm^3^) determined in case of cell-cell and cell-ECM in molecular-level simulations results in Fig. S5b contribute to the overall cohesiveness in the molecular-level models. This phenomenon can also be visualized in Fig. 5c, where van der Waals surfaces have been generated for the clay crystallites sorbed on the cell-ECM complex in Fig. S8e. Sorption of clay crystallites in cell-ECM structure forms closely connected and overlapping strong van der Waals attraction fields. These attraction fields formed by van der Waals energies have resulted in higher cohesive energy density of the clay-cell-ECM configuration. Based on their ZP, Na-montmorillonite nanoparticles are hydrophilic in nature, while cancer cells and fibronectin are hydrophobic in nature (Table S4). Although generally, repulsions are expected among hydrophilic and hydrophobic surfaces in deionized water, in contrast^45^, discovered a typically DLVO-like interaction between hydrophobic polystyrene particles and a hydrophilic surface generated by the addition of salts. In the presence of salts, secondary adhesion processes were observed between hydrophobic and hydrophilic surfaces^46^. Accordingly, in RPMI medium, similar phenomenon as discussed in^46^ occurs between Na-montmorillonite particles and the cancer cells. It is also worth noting that the hydrophilic nature and high repulsive acid-base (AB) interactions (Table S3) of these clay particles results in more dispersion. High dispersion, in turn, leads to the availability of high surface area for the enhancement of the attractive interactions. As mentioned earlier, flaky equidimensional particles of Na-montmorillonite have, therefore, a tendency to form larger particles by flocculating at the edges once these precipitate from the saturated RPMI solution. These bigger particles bind and completely cover/bridge across the multiple cells as shown in SEM (Fig. 6Aa) and the corresponding sketch in Fig. 6Ab. This mechanism is not only quite effective in restoring the specific adhesion among Raji cells but also in restraining the movement of the cells from their primary location. Although of lesser degree, positively charged edges of Na-montmorillonite particles, also get electrically associated with the cancer cells. All these phenomena are a testimony of higher cell-cell adhesion recorded in AFM testing for Na-montmorillonite (Fig. 3a).

Like Na-montmorillonite, hectorite, do flocculate at edges and ends, but due to their relatively lesser dispersion capacity (ZP = −29 mV) and elongated/rectangular particle shape (Fig. 1b**)**, do not form bigger particles. Therefore, as observed in Fig. 6B, hectorite, due to their limited particle sizes, are not able to completely cover and overlap the Raji cells. Similarly, palygorskite nanoparticles, due to their ZP of −24.2 mV, are mildly hydrophobic in nature. Due to their mild hydrophobicity and high attractive AB interactions, palygorskite particles have the tendency of agglomeration in the colloidal form. The palygorskite agglomerates are, therefore, not quite effective in creating complete coating and bridging of the cancer cells, and thus do not enhance adhesion among cancer cells to the same degree as Na-montmorillonite.

Contrary to their behavior in the enhancement of cell-cell adhesion, hectorite and palygorskite have been found to be better in the restoration of adhesion among cancer cells and ECM proteins (Raji-FN in Fig. 3b). One of the apparent reason of this behavior is the similarity of the elongated/thread-like particles of hectorite and palygorskite to the active integrins bridging and binding the healthy cells to the ECM proteins. Moreover, relatively higher surface area along the surfaces and edges contribute higher level of van der Waals interactions with FN. Therefore, due to their particular shape and the charged surfaces and edges, these nanoparticles have been found to be more effective than Na-montmorillonite in bridging the inactive integrins and binding the cancer cell surfaces to FN. This phenomenon is also quite obvious in the corresponding SEMs (Fig. 2c and d) where thread-like and elongated particles seem to be well-embedded and anchored in FN. As envisioned in the SEM results, due to relatively smaller particle sizes, palygorskite remained effective in the non-specific receptor mediated van der Waals and hydrophobic-hydrophobic binding of cell-ECM, while it has been found to be less effective in binding cell-cell assemblies where Na-montmorillonite has taken the lead.

### Combined Na-montmorillonite and palygorskite nanoparticles model

The results of the combined influence of different proportions of a mixture of Na-montmorillonite and palygorskite on a cell-cell-ECM assembly (Fig. 3c) indicate that the maximum increase in the adhesion of the cancer cell environment occurred at a 75/25 proportion of Na-montmorillonite and palygorskite. On the other hand, 50/50 and 25/75 proportions have resulted only in slight increase in adhesion than the untreated/control assembly. This phenomenon could be explained with respect to the relative surface area, flocculation and dispersion tendency, interlayer charges, particle size distribution, and the specific interaction with cancer cells and ECM of both the clays. It could be observed from Table S1 that Na-montmorillonite and palygorskite have the same surface area when mixed in a ratio of 75/25. Having the same surface area at this mix ratio, both the clays seem to be interacting with each other in an optimum manner. This same surface area effect at 75/25 proportion, could further be visualized from SEMs in Fig. 2h and k, where one can notice that the particles resulting from 75/25 proportion are almost equally proportioned from Na-montmorillonite flaky particles and the thread-like palygorskite particles. On the other hand, due to quite higher surface area of palygorskite (4.3 and 12.8 times in 50/50 and 25/75 proportioned mixtures), it completely envelopes and create thick layers on the Na-montmorillonite particles (SEMs in Fig. 2f,g,i, and j). In addition to the equivalency in surface area, the interlayer charges (Table S1) of palygorskite and Na-montmorillonite also become equal at a proportion of 75/25. This fact further validates the concept of an optimum interaction of both the clays at this proportion. As discussed in the previous sections, palygorskite itself is relatively inferior in promoting cell-cell adhesion and with its thick coating on Na-montmorillonite, it is preventing the latter to promote cell-cell adhesion. In the case of Na-montmorillonite alone, although clay particles are completely coating and adhering to the cells, there is a reduction in the adhesion compared to when palygorskite is present as 25% proportion of the clay mixture. This reduction in adhesion for the case of Na-montmorillonite alone is most probably due to the lack of adhesion in the cell-ECM assembly otherwise would have been promoted by palygorskite. Another reason for the least contribution of the 50/50 and 25/75 proportions is relatively much smaller united particle size of D_50_ = 34 and 25 nm) as compared to the mean size (D_50_ = 49 nm) of the 75/25 mixture. Use of an optimum combination of Na-montmorillonite and palygorskite clays to maximize their properties is also quite common practice in the industry for several purposes. In a study of the use of a mixture of these two clays for the removal of mycotoxins^47^, the authors discovered that an almost equal combination of montmorillonite and palygorskite provided maximum removal of the toxins. The SEMs provided in their study reveal that the optimum combination of these two clays is achieved when montmorillonite is completely covered by palygorskite. In another study^48^, the rheology/viscosity of a mixture of palygorskite-montmorillonite was also found to be minimum at montmorillonite percentages of approximately 40–50%, while resistance to flow was maximum at higher percentages of montmorillonite. Nano-sized palygorskite was also found to be quite effective in tailoring and modification of the viscosity of the drilling mud (Na-montmorillonite)^49^. In the same study, the authors discovered that addition of an optimum percentage of palygorskite to montmorillonite also results in the reduction of the combined/united particle size of the two clays. From the results of all these studies, it could be inferred that lower viscosity in the case of an optimized 75/25 mixture of Na-montmorillonite and palygorskite compared to the more viscous mixtures of 50/50 and 25/75 leads to an effective coating and hence better adhesion among the Raji cells-FN.

## Conclusions

This study has demonstrated through adhesion measurements using AFM and molecular-level simulations that the high affinity of the charged clay nanoparticles to the charged cancer surfaces, combined with the increased non-specific adhesion affinity of cancer cells, makes these clay nanoparticles potentially promising candidates to adhere to the cancer cells and hence control metastasis. The underlying idea of this novel approach is that, once bound to clay particles, cancer cells would be constrained from migrating to other parts of the body, thereby allowing more effective local therapeutic treatments.

The AFM measurements demonstrate that maximum increase in cell-cell adhesion was achieved with Na-montmorillonite, while palygorskite and hectorite have resulted in a maximum increase in the case of cell-FN interactions. The combined effect of Na-montmorillonite and palygorskite on the cell-cell-FN assembly demonstrated 75/25 as the most effective proportion resulting in a maximum increase in adhesion. Scratch-induced wound healing assays also confirmed the adhesive nature of the clay particles in controlling the migration of the cancer cells.

Clay nanoparticles join with each other at their edges/ends to form bigger particles that would envelope and adhere to the multiple cancer cells and their environment mainly through electrostatic, van der Waals, and the zeta potential (ZP) interactions among individual cells and the clay nanoparticles. Clay nanoparticle flocks would also provide the bridging across the multiple cancer cells.

Based on the nature and extent of the surface properties, and the configuration of the cancer cells, ECM, and the clay particles as ascertained from the tests and SEMs in this research and the previous research, interaction models of Na-montmorillonite, hectorite, and palygorskite with the cell-cell-ECM environment have been developed. In each of the interaction models, several modes of interactions have been identified constituting of attractions due to electrostatic and van der Waals forces, ZP electrostatic interactions, and bridging between cell-cell and cell-FN. These modes of interaction among the clay nanoparticles and cancer cells and their environment contributes to the overall adhesiveness of the system in various forms and to different degree.

These encouraging results support the commencement of *in vivo* studies to investigate the practical applicability of the approach. Based on the conclusions of the current study, *in vivo* research has been planned using human melanoma xenograft animal models using 75/25 proportion of Na-montmorillonite and palygorskite^50–63^.

## Supplementary information

Materials and Methods
