# Supplementary material for: Enhancing cancer cell adhesion with clay nanoparticles for countering metastasis
Source: Sci Rep. 2019 Apr 11;9:5935. doi: 10.1038/s41598-019-42498-y (PMC6459834; doi:10.1038/s41598-019-42498-y)

Enhancing cancer cell adhesion with clay nanoparticles for countering metastasis

Sahel N. Abduljauwad^1^ and Habib-ur-Rehman Ahmed^1^*

^1^Civil & Environmental Engineering Department, King Fahd University of Petroleum & Minerals (KFUPM), Dhahran, Saudi Arabia

*Correspondence to: habibrehman@kfupm.edu.sa

*Supplementary material*

**MATERIALS AND METHODS**

**MATERIALS**

***Cancer cells and ECM protein samples***

The Raji cell line was obtained from the American Type Culture Collection (ATCC) as CCL-86. Raji cells were maintained in RPMI-1640 medium (Thermo Fisher Scientific) supplemented with 10% fetal bovine serum (FBS) (Atlanta Biologicals) and penicillin/streptomycin (50 U/mL/50 μg/mL CellGro) in 5% CO2 at 37°C. Cells were passaged every 72 hours and 12–24 hours before measurements. The basic characterizations of these cells and FN relevant to the current study are summarized in **Table S4**.

To represent ECM, human plasma fibronectin (FN) samples were obtained from Sigma. FN was stored at a temperature of 2-8^o^C before being used for the cell culture.

***Nanoparticle isolation and purification***

Na-montmorillonite (SWy-3), hectorite (SHCa-l), palygorskite (PFl-l), kaolinite (KGa-1) clay samples were acquired from the Clay Minerals Society (CMS) [36, 37] (**Tables S1 and S2**). Nano-sized particles of pure clay minerals were obtained by a multi-step procedure involving the deflocculation of the initial dry powdered state and the removal of non-clay minerals and impurities. Most of the steps followed the general guidelines provided in ASTM D7928 [53]. First, nanoparticle aggregates in each clay sample were broken down by mixing 5 g of sample with 12.5 ml deflocculant solution (40 g sodium hexametaphosphate in 1000 ml of deionized water) and leaving for 16 hours for uniform absorption. Next, the mixture was diluted to 100 ml using deionized water and centrifuged in a standard cup at a speed of 1000 rpm for 30 minutes. The supernatant was poured into a glass cylinder and further diluted to 1000 ml. The mixture then stood for sedimentation under gravity to remove bigger aggregates and non-clay particles for a period of 12 hours. The upper 500 ml portion of the colloid suspension was then extracted.

***Nanoparticle size and purity analysis***

The clay colloid size distributions were analyzed by diffraction laser system (DLS). We used a Microtrac S3500 DLS instrument, comprising a Tri-laser system operating at 780 nm wavelength and capable of detecting particle sizes in the range of 0.02 µm to 2.8 mm. The run time of each analysis was less than 60 seconds. The data acquired in the test consisted of particle sizes and their frequency in the suspension. This data was converted by the acquisition software (FLEX) of the instrument to a statistical distribution in the form of the particle size and the corresponding percent passing. The statistical data is then converted to percentile and the corresponding particle size.

Size distributions were determined both in deionized water and RPMI 1640 medium. The purpose of doing particle size distribution in deionized water was to determine the maximum possible dispersion of the particles. The resulting finest gradation of each of the clays in deionized water, was then compared with the one obtained in RPMI medium. The purpose wasto assess the effect of presence of salts in RPMI on the flocculation / agglomeration tendency of the clay particles.

After DLS testing, the dry sample of each of the clays was recovered from the colloidal form by evaporating the mixture in an oven at 110°C for 24 hours. The purity of the recovered clay mineral was assessed by X-ray diffraction (XRD). The XRD results for the natural and the pure clay samples are presented in **Fig. S1**, while the corresponding particle size distribution plots are shown in **Fig. S2**.

**METHODS**

**Clay suspension preparation**

The purified clay samples were suspended in RPMI 1640 medium. Initial trials showed that the particles could not be suspended at high concentrations even when left overnight after mixing. Stock saturated suspensions (0.2 mg/ml) of the clay particles were thus prepared in RPMI medium. The particle size distribution was measured by DLS as detailed above.

**Clay nanoparticles cytotoxicity verification**

To eliminate any possibility of toxic effect of the clay particles on the cancer cells, before conducting the adhesion tests, we verified the possible toxicity of the clay nanoparticles in the cancer cells. The clay particles were suspended in sterilized medium to eliminate any contamination by pathogens. To sterilize the medium for cell culture, the cleared clay solution of 0.2 mg/ml was autoclaved for 30 min at 121°C and a pressure of approximately 30 psi. The cancer cells were then exposed to the sterilized clay suspension for one week to assess the cytotoxicity. The cell vitality results after treatment with various proportions of clay nanoparticles indicate that the clay particles do not result in the retardation of the growth / proliferation of the cancer cells and these remain viable (98 – 100%) during this period.

**AFM measurements**

AFM measurements were carried out using an Asylum Research MFP-3D-BIO AFM (Goleta, California, US) mounted on a Nikon A1 confocal microscope at the Miller School of Medicine, University of Miami, Florida. All measurements were carried out at room temperature (25ºC) at a scan velocity of 2 μm/sec. The force measurements were carried out using a Veeco MLCT-O10 tipless cantilevers (Camarillo, California, US) with a nominal spring constant of 0.01 N/m. AFM adhesion measurements were conducted using the principle schematically shown in **Fig. S3a and S3b**. The measurements were performed on 3 different configurations (cell-FN, cell-cell, and cell-cell-FN) shown in **Fig. S3c**.

***Raji cell to fibronectin***

Cantilevers were initially salinized with 3-aminopropyltriethoxysilane. After incubation of the cantilevers with 0.1% glutaraldehyde for 30 min, FN (2.5 μg/ml) were coupled to the cantilever through the glutaraldehyde linker. Incubation for 1 h with 1% bovine serum albumin (BSA) was used to block the bare surfaces of the cantilever.

Raji cells were plated on 35 mm Petri dishes (Falcon 351008) that were coated overnight with 0.1 mg/ml poly-L-lysine (Sigma) and returned to the incubator for 30 min to allow the cells to get attached to the surface of the dish (**Fig. S3c-A**). Afterwards, the cells were exposed to clay suspensions (PFl-1, SHCa-1 or SWy-3) at the concentration of 0.2 mg/ml. After 30 min, the clay medium was replaced with fresh cell culture medium. With the aid of the light microscope, the end of a fibronectin-functionalized cantilever was positioned on the center of a Raji cell. Adhesion measurements were acquired by lowering the cantilever onto the Raji cell and withdrawing the cantilever until it detaches from the cell. Measurements were averaged for three trials; each trial in turn consisted of 15 measurements on 15 cells. Typical AFM measurements consisting of the plot of cantilever approach and the attachment and subsequent detachment plots for the interactions are shown in **Fig. S4**.

***Raji cells interactions***

For cell-cell measurements, the cantilevers were functionalized with concanavalin A (ConA). For the purpose, the cantilevers were soaked briefly in 0.1 M NaHCO3 (pH 9.0) to ionize the surface of the cantilevers, removed from the NaHCO3 solution, air-dried, and immersed in 100 μl of ConA (0.1 mg/ml in PBS buffer) overnight at 4°C.ConA–functionalized tipless AFM cantilevers were then further functionalized with Raji cells using a glutaraldehyde linkage [54].

The measurements were made between Raji cell attached to the end of the AFM cantilever and a Raji monolayer plated on a tissue culture dish, without and with the clay suspension in RPMI medium at a concentration of 0.2 mg/ml. The measurements were repeated in the absence and in the presence of the charged clays (Na-montmorillonite, hectorite, and palygorskite) and of the uncharged / neutral clay kaolinite.

***Raji cells and FN interactions***

Based on the initial findings of the relatively better enhancement of cell-cell adhesion by SWy-3 and cell-FN adhesion by PFl-1, it was decided to study the combined effect of these two clays on a combined assembly of cell-cell-FN.

AFM measurements were made on the adhesion induced by 25/75, 50/50, and 75/25 proportioned mixtures of PFl-1/SWy-3.These proportions correspond to 0.05/0.15, 0.1/0.1, and 0.15/0.05 mg/ml concentrations of PFl-1 and SWy-3respectively.Raji cells were cultured on 35 mm Petri dishes (Falcon 351008) dish coated with fibronectin overnight. As explained earlier, Raji cells were attached to the AFM cantilever using poly-L-lysine. The AFM force measurements were carried out in RPMI medium supplemented with fetal bovine serum and 20 µg/ml fibronectin.

***Statistical analysis***

GraphPad Prism 8 program was used for statistical analysis. A one-way analysis of variance (ANOVA) was used to identify a difference followed by Student T-tests. P-values of less than 0.05 were obtained in the analysis and considered statistically significant.

**Scratch induced wound healing assays**

To assess the role of clay nanoparticles in the cell adhesion and migration, a wound healing assay was carried out on MCF7 cells.  For the purpose, MCF7 cells were cultured in a tissue culture treated 8 well microscope slide (Ibidi USA, Inc.) in RPMI medium supplemented with 10% fetal calf serum until confluent (**Figs. S5 and 4**). A glass pipette was used to scratch a gap of approximately 50 microns in the cell monolayer.  Cultured medium was exchanges with fresh medium with or without clay nanoparticles.  Cells were returned to 37°C incubator and cultured for 24 hours.  After which, cells were images to assess the extent of cell migration in the scratch region. Image analysis was carried out to measure percent closure of the scratched area within the captured images.

**Scanning electron microscopy (SEM)**

Before imaging, samples were preserved in 2% glutaraldehyde fixative in PBS buffer and stored in the refrigerator for at least 2-3 hrs. Samples were washed in three changes of PBS buffer for 10 min each and were then post-fixed in 1% osmium tetroxide in PBS buffer for 45 mins and then rinsed in three changes of PBS buffer for 10 mins each. The cells were then dehydrated in a graded series of ethanol (20, 50, 70, 95, and 100%). After dehydration, samples were dried in three changes of HMDS and left to outgas overnight.

Cells on cover slips were then placed on aluminum stubs covered with carbon adhesive tabs. Pelleted cells were placed directly on the carbon adhesive tabs on the stubs.

Sample imaging was performed in SEM mode in an FEI ESEM-FEG XL-30 at the Miller School of Medicine, University of Miami, Florida.Images at various magnifications were acquired to reveal the different types of clay particles covering the individual cells and bridging the multiple cells. The images were also obtained focusing on the micro-interaction of individual and mixture of clay particles with cells and the FN.

**MOLECULAR-LEVEL SIMULATIONS STUDY**

This part of the study consisted of the creation of cell-cell and cell-ECM configurations in a molecular simulation software followed by the simulations of the interactions with clay crystallites of various configurations and levels of activity, including those obtained from CMS [36]. A cancer cell-ECM configuration consisted of a plasma membrane with an integrin surrounded by several proteins of ECM. The inactive integrin was used to signify the cancer cell. Similarly, cell-cell configuration was created using two plasma membranes with the attached cadherin. Although these models may not be the complete representation of the actual in vitro conditions, these have been incorporated with all the essential interaction components of a cell and are well suited for the intended relative comparative studies. The sorption and simulations of Na-montmorillonite, hectorite, palygorskite, and kaolinite clay crystallites in the formulated configurations were carried out using Monte Carlo (MC), and molecular mechanics (MM) techniques in Materials Studio software [55]. The enhancement of adhesion in all the simulated configurations was assessed in terms of the calculated cohesive energy density (CED), considered as a measurement of the cohesiveness of the molecular system. Total CED of each of the configuration was compared qualitatively with the adhesion achieved during AFM measurements, while van der Waals and electrostatic components of CED were used to explain various levels of adhesion measured for different clays in AFM. Due to the large volume of computations involved in the simulations, these calculations were carried out using the high-performance computing facilities (HPC) at KFUPM, KSA. The overall methodology and the choice of individual methods and the simulation parameters were based on authors′ previous research [38-44].

***Selection and formulation of clay crystallites***

Unit clay molecules used in the formulation are Na-montmorillonite, hectorite, palygorskite, and kaolinite crystallites. These were formulated based on the basic properties summarized in **Tables S1 and S2** such as CEC, exchangeable cations, and the interlayer charges. The size of the molecular / crystallite size is selected based on the results of the particle size analysis using the DLS technique. Using the particle size distribution results (**Fig. S2**), the mean particle size of each of the clays is tabulated in **Table S5**. Using these mean particle sizes, the calculated dimensions of the crystallite size required to be used in the simulations are also listed in **Table S5**. Typical molecular / crystallite models of Na-montmorillonite, hectorite, and palygorskite are shown in **Figs. S6a** to **S6c**. After preparation of these crystallites in the design mode of the software using the data in **Tables S1** and**S2**, these were charged using the charge equilibration method QEq of the software.

***Formulation of cell-ECM and cell-cell configurations***

The cancer cell-ECM model consisted of plasma membrane with an embedded integrin and surrounded by ECM proteinmolecules, while a cell-cell adhesion configuration was created using the ends of cadherin sandwiched between two parts of plasma membranes on both sides. Molecules of integrin, cadherin, and other associated ECM proteins such as laminin, collagen, and fibronectin were mainly acquired from the protein data bank websites RCSB and PDB-101 [56, 57]. Additionally, plasma membrane files in protein data bank (PDB) format were acquired from the University of Calgary website [58].

The integrin was created using three different parts formulating an inactive integrin. The top structure of the integrin that makes the extracellular portion extend outward from the cell surface (PDB entry 1jv2) was connected through the membrane by a short transmembrane section (PDB entry 2k9j) and the two short cytoplasmic tails extend into the cell (PDB entry 1m8o). Similarly, cadherin, in the form of large proteins that extend from the surface of the cell, was obtained from PDB entry 1l3w.

For the formulation of cell-ECM configuration (**Fig. S7a**), the tail of the inactive integrin was sandwiched between two parts of the plasma membrane, and the rest protruded out towards the ECM proteins. In this configuration, ECM was mimicked using three main proteins, i.e., collagen, laminin, and fibronectin. Similarly, a cell-cell adhesion configuration was created using the ends of cadherin sandwiched between two parts of plasma membranes on both sides; the software generated unit cell is shown in **Fig. S7b**. After placing the cell-cell and cell-ECM components within their relative positions and distances, the entire geometry was optimized by lowering the energy using the geometry optimization option of the Forcite module of the software. The Forcite geometry optimization task refines the geometry of a structure using an iterative process, in which the atomic coordinates, and the cell parameters, are adjusted until the total energy of the structure corresponds to a minimum in the potential energy surface. In this study, the magnitude of forces on the atoms calculated using modified universal forcefield [38] were reduced until they become smaller than the defined convergence force tolerance of 0.5 kcal/mol/Å. The Smart algorithm, consisting of a cascade of the steepest descent, ABNR, and quasi-Newton methods, was used in this study. Combination cascade provides a sequential lowering of the minimum potential energy of the system.

***Clay crystallite interactions with cell-ECM and cell-cell configuration***

Since the required crystallite sizes in **Table S5** were too big to be computationally handled even at high-performance computing facilities, different combinations of the size and number of the crystallites of each type of clay were used in the simulations. From the results, it was discovered that cohesive energy density (CED) remain the same for the equivalent surface area of the any combinations of size and number of the specific crystallites. This fact resulted in allowing the use of the smaller-sized crystallites (**Table S5)** in the simulations. The resulting CED per unit surface area of a specific number and size of crystallites were then projected to the surface area of the required size and number of crystallites. Therefore, to simulate the interaction of clay crystallites with cell-cell and cell-ECM complexes, various sizes and numbers of the crystallites of different clays (**Table S5**) were sorbed on each cell-ECM and cell-cell configurations using the Sorption module of the software. For the simulations, the Metropolis Monte Carlo method was selected in the Sorption module of the software. In each sorption step, clay crystallites occupied spaces in the unit cell to lower the overall energy of the complex. The required number of crystallites were sorbed in a maximum of 25000 steps, and then the energy of the system was minimized using the Forcite module of the software based on the MD principles. The Forcite module of the Materials Studio software with the NPT (constant number of particles, pressure, and temperature) ensemble was used, and simulations were performed using a modified universal force field for 5 to 30 ps in 0.5-fs intervals or until a constant volume was reached. It was observed during the simulations that the lowest energy configuration was achieved within a maximum simulation period 30 ps. A Berendsen thermostat with a decay constant of 0.1 ps was used to control the temperature during the simulation. During the MD simulation, the assumed temperature was kept constant at 310K (37°C). Simulations were carried out assuming an atmospheric pressure (100 kPa), and a Berendsen barostat with a decay constant of 0.1 ps was used to control the pressure of the system. The Berendsen methodology was selected as the most suitable for the single crystallites after several trials involving other thermostats and barostats available in the software. In the Monte Carlo method, the parameters for the ratios of exchange, conformer, rotate, translate, and regrow were selected as 0.39, 0.2, 0.2, 0.2, 0.2 respectively, while the corresponding probabilities were 0.39, 0.2, 0.2, 0.2, and 0.2. Amplitudes adapted for rotation and translation were 5° and 1 Å, respectively. The final configurations after sorbing crystallites of various sizes and numbers of Na-montmorillonite, hectorite, and palygorskite crystallites on the cell-ECM and cell-cell complexes are shown in **Fig. S8.**

***Cohesive energy density (CED) measurement***

In this study, the assessment of the adhesion created by the clay crystallites in the cell-cell and cell-ECM complexes was made through the changes in CED. After the sorption of clay crystallites and the subsequent molecular dynamics of each of the configurations, the CED was determined using the Cohesive Energy Density option of the Forcite module of the software. The authors have experienced that the CED concept, consisting of total, van der Waals and electrostatic CEDs, can quite closely explain the various molecular-level processes and interactions and simulate the extent of adhesion/binding created among the simulated complexes [59-63]. Quantitatively, CED is the amount of energy needed for the transition of 1 mol of material from the liquid to the gaseous phase. It is considered as a measure of the mutual attractiveness of molecules, being expressed as a combination of electrostatic and van der Waals forces, averaged over an NPT ensemble. Further details can be found in [38-44].

In the Forcite module, van der Waals energies were evaluated using atom-based cutoffs. In this method, non-bond interactions are simply calculated to a cutoff distance and interactions beyond this distance are ignored. To avoid the discontinuities caused by direct cutoffs, most simulations use some kind of switching function to smoothly turn off non-bond interactions over a range of distances. An effective potential is created by multiplying the actual potential by the smoothing function. The choice of the function in the intermediate range is crucial and should be continuously differentiable in this region so that forces can be calculated. In this study, a cubic spline smoothing function has been used with a spline width of 1 Å and cutoff distance of 12.5 Å.

For comparison purposes, the electrostatic and van der Waals CEDs of various cell-ECM and cell-cell and cell-ECM complexes with and without interactions with various clay crystallites are plotted in **Fig. 4a and 4b**.

**Table S1: Summary of chemical and physical characterizations of clay samples [31**]

| **Sample Designation** | | **Clay mineral** | | **Other minerals** | | **Surface Area N_2_ (m^2^/g)** | | **CEC (meq/100 g)** | **Exchangeable cations** | | **Octahedral charge** | | **Tetrahedral charge** | **Interlayer charge** |
| --- | --- | --- | --- | --- | --- | --- | --- | --- | --- | --- | --- | --- | --- | --- |
| **SWy-3** | **Na-montmorillonite** | | **5% silica** | | **31.82** | | **76.4** | | | **Na, Ca** | **-0.53** | **-0.02** | | **-0.55** |
| **SHCa-1** | **Hectorite** | | **43% calcite 3% dolomite** | | **63.19** | | **43.9** | | | **Ca, Mg** | **-1.35** | **-0.22** | | **-1.57** |
| **PFl-1** | **Palygorskite** | | **5% silica** | | **136.15** | | **19.5** | | | **Mg** | **-1.87** | **-0.22** | | **-2.09** |
| **KGa-1** | **Kaolinite** | | **5% silica** | | **10.05** | | **2.0** | | | **-** | **0.11** | **-0.17** | | **-0.06** |

**Table S2: Summary of chemical composition of the clay samples [36**]

| **Sample Designation** | | **Major clay mineral** | **Source** | **Chemical formula** |
| --- | --- | --- | --- | --- |
| **SWy-3** | **Na-montmorillonite** | | **Crook County, WY, USA** | **(Na,Ca)_0.33_(Al,Mg)_2_(Si_4_O_10_)** |
| **SHCa-1** | **Hectorite** | | **San Bernardino County, CA, USA** | **Na_0.3_(Mg,Li)_3_Si_4_O_10_(OH)_2_** |
| **PFl-1** | **Palygorskite** | | **Gadsden County, FL, USA** | **(Mg,Al)_2_Si_4_O_10_(OH)_4_H_2_O** |
| **KGa-1** | **Kaolinite** | | **Washington County, GA, USA** | **(Mg_.02_,Ca_.01_,Na_.01_,K_.01_) Si_3.83_Al_.17_O_10_(OH)_8_** |

**Table S3: Summary of chemical and physical characterizations of clay samples [37**]

| **Sample Designation** | **Clay mineral** | **Zeta potential (ZP)**  **(mV)** | **γ_s_^-^** | **Water affinity** | **Interaction energy**  **(AB)** | **Interaction energy (vdW)** | **Interaction energy (Total)** | **Flocculation/ Dispersion in water** |
| --- | --- | --- | --- | --- | --- | --- | --- | --- |
| **SWy-3** | **Na-montmorillonite** | **-31.9** | **44.6** | **Hydrophilic / Polar** | **22400** | **-730** | **22800** | **Dispersion** |
| **SHCa-1** | **Hectorite** | **-29.7** | **20.7** | **Mildly Hydrophobic to Hydrophilic** | **-5070** | **-730** | **-4800** | **Flocculation / Dispersion** |
| **PFl-1** | **Palygorskite** | **-24.2** | **23.2** | **Mildly Hydrophobic** | **-3100** | **-230** | **-2690** | **Flocculation** |
| **KGa-1** | **Kaolinite** | **-49.2** | **30.0** | **Highly Hydrophilic / Polar** | **5600** | **-730** | **7510** | **Flocculation** |

**Table S4: Summary of electrical properties of cancer cells and fibronectin [50-52**]

| **Cell / ECM sample** | **Description** | **Zeta potential (ZP)**  **(mV)** | **Water affinity** | **Net charge on the surface** | **Flocculation/ Dispersion in water** |
| --- | --- | --- | --- | --- | --- |
| **Raji** | **Lymphoma cancer cell line** | **-17.0** | **Mildly Hydrophobic** | **Negative** | **Flocculation** |
| **FN** | **Human plasma ECM protein** | **-11.0** | **Moderately Hydrophobic** | **Positive** | **Flocculation** |

**Table S5: Summary of mean particle size measured by diffraction laser system** (DLS) and the corresponding crystallite sizes of the clay minerals adopted in molecular-level simulations

| **Sample Designation** | **Clay mineral** | **Mean size from DLS (Å)** | **Size required in molecular simulations (Å)** | **Surface area required (Å^2^)** | **Size(s) adopted (Å)** | **Surface area adopted (Å^2^)** | **Equivalent number of crystallites** |
| --- | --- | --- | --- | --- | --- | --- | --- |
| **SWy-3** | **Na-montmorillonite** | **480** | **501x501x20** | **542082** | **150x150x20**  **108x26x20** | **57000**  **10976** | **10**  **50** |
| **SHCa-1** | **Hectorite** | **510** | **501x50x20** | **72140** | **520x54x20**  **108x26x20** | **79120**  **10976** | **1**  **7** |
| **PFl-1** | **Palygorskite** | **270** | **276x26x26** | **30056** | **317x27x27**  **127x14x27** | **35694**  **11170** | **1**  **3** |


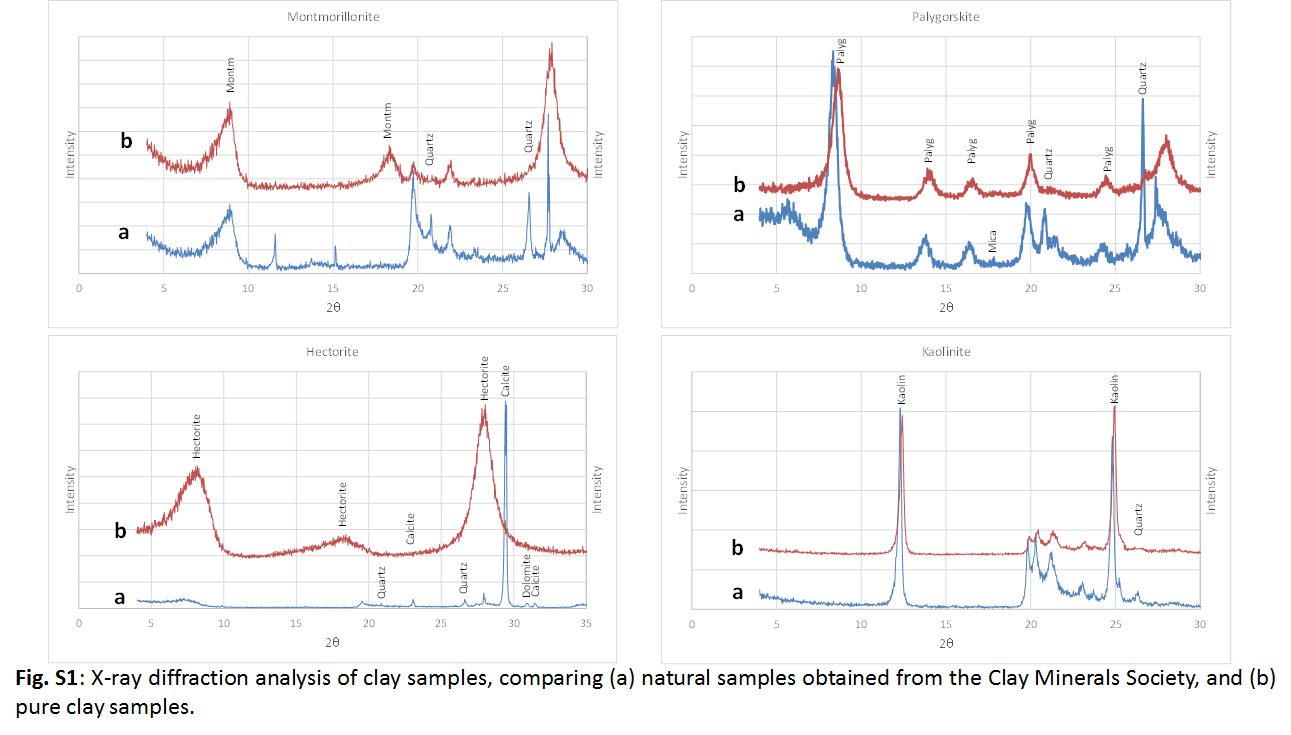


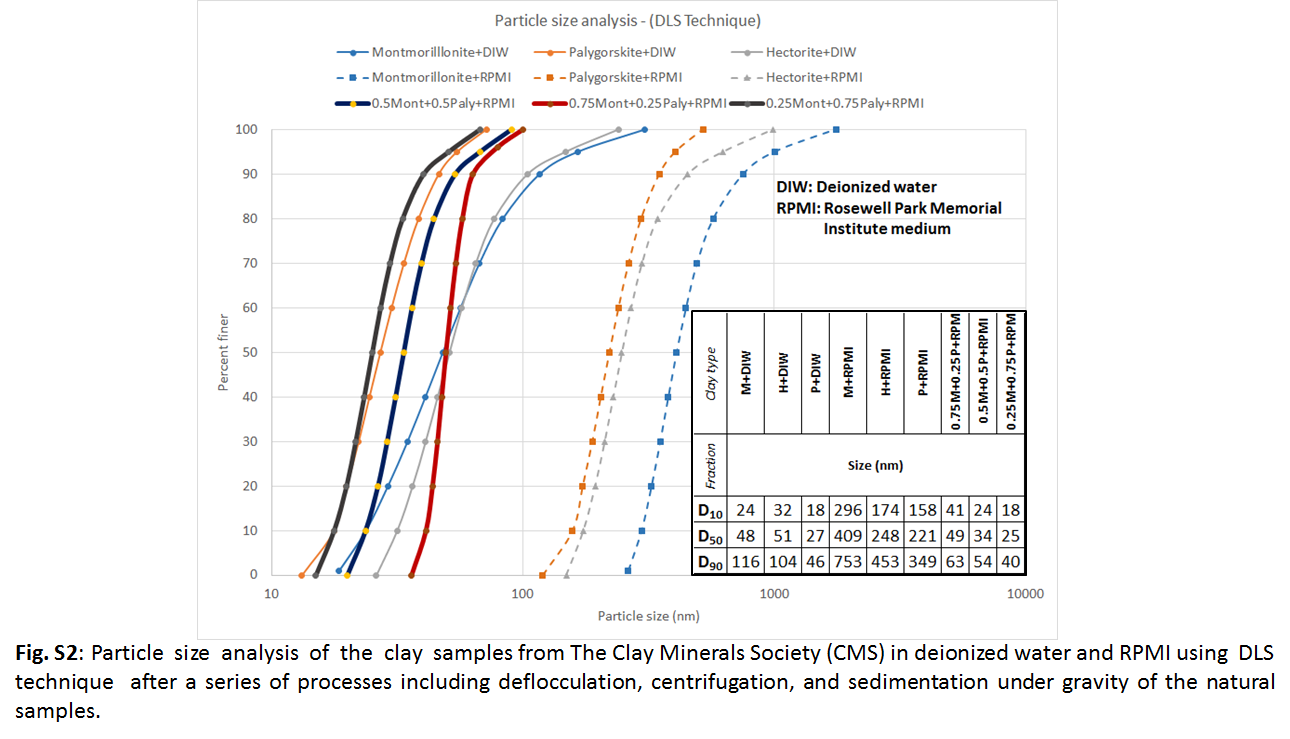


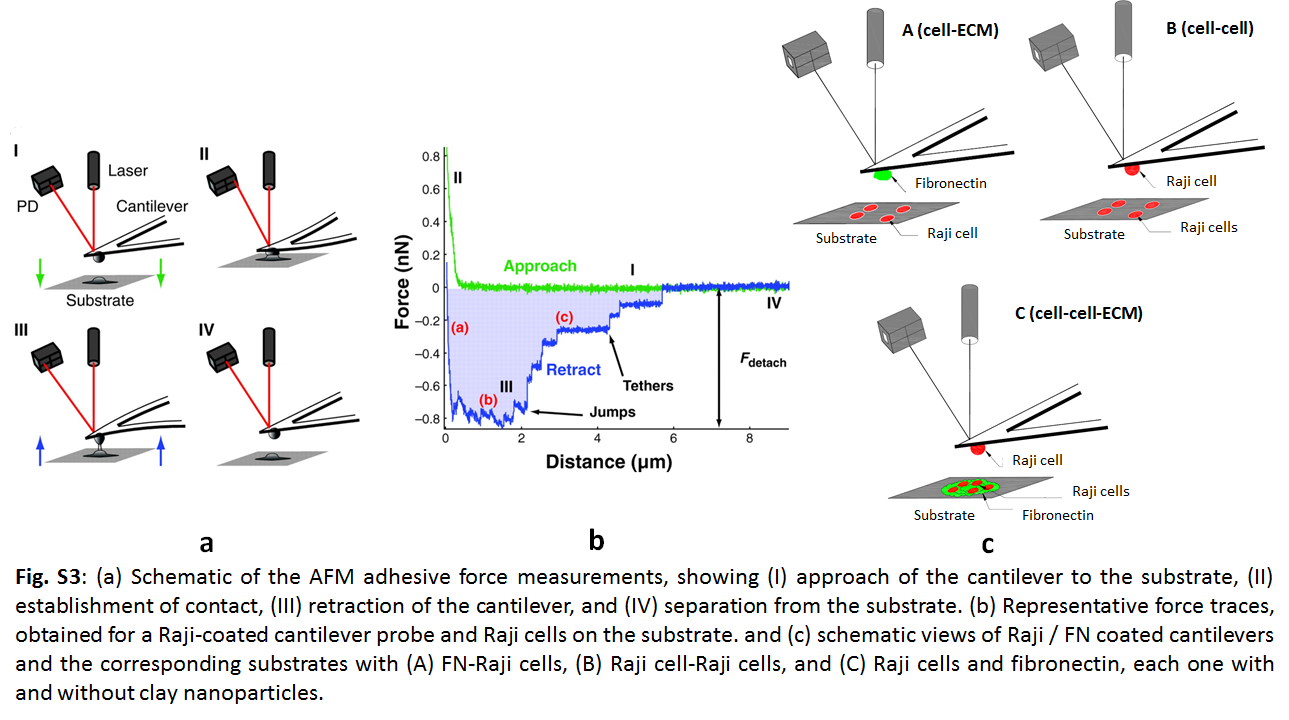


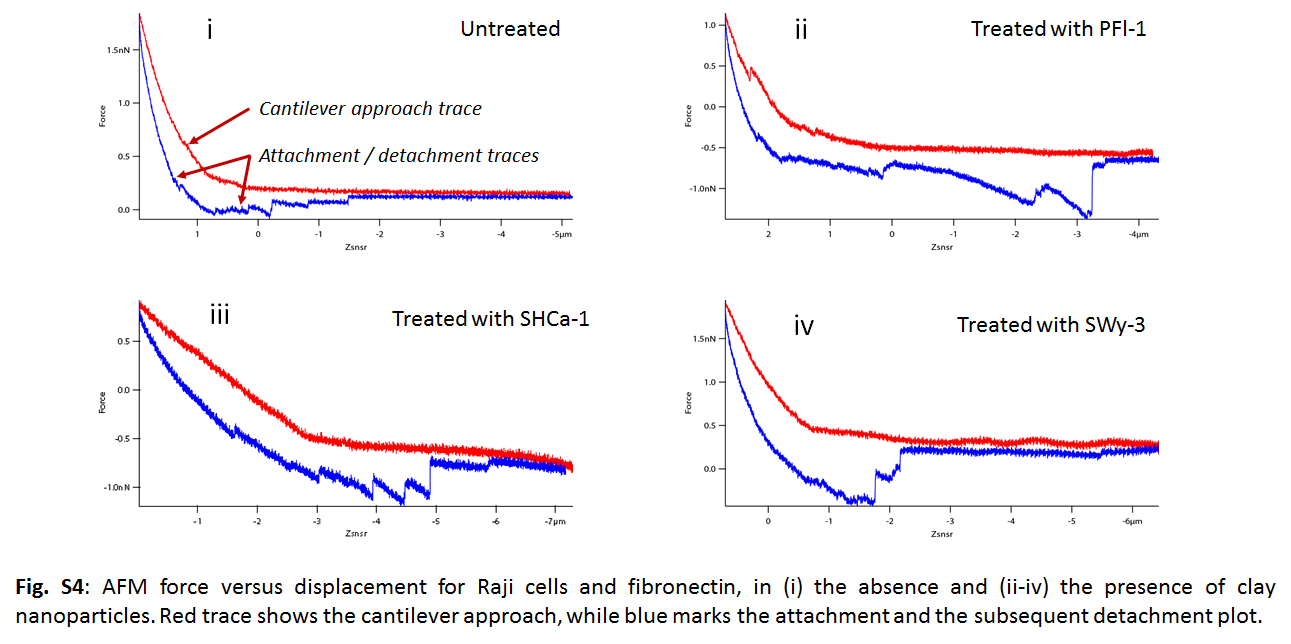


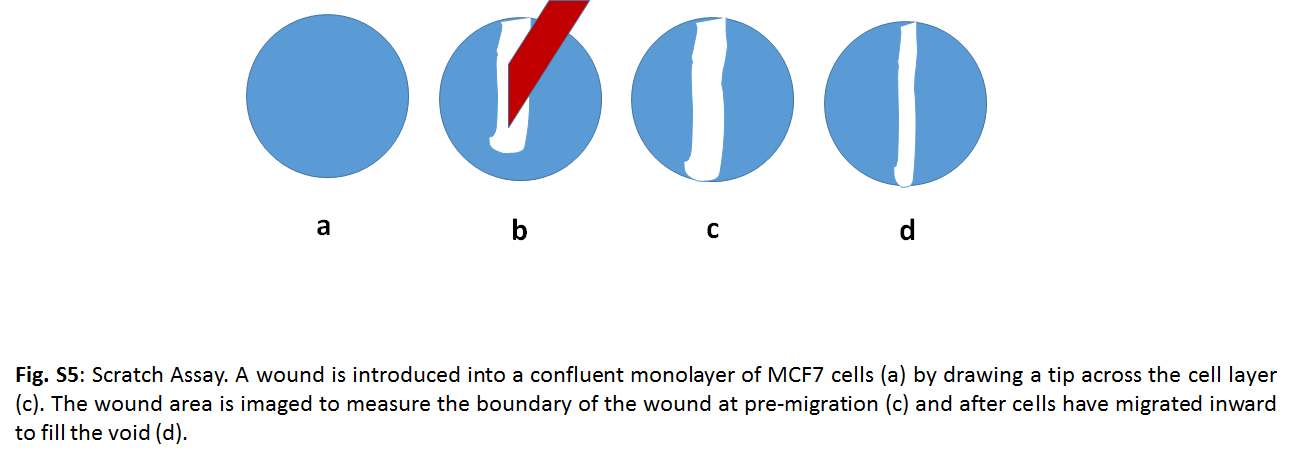


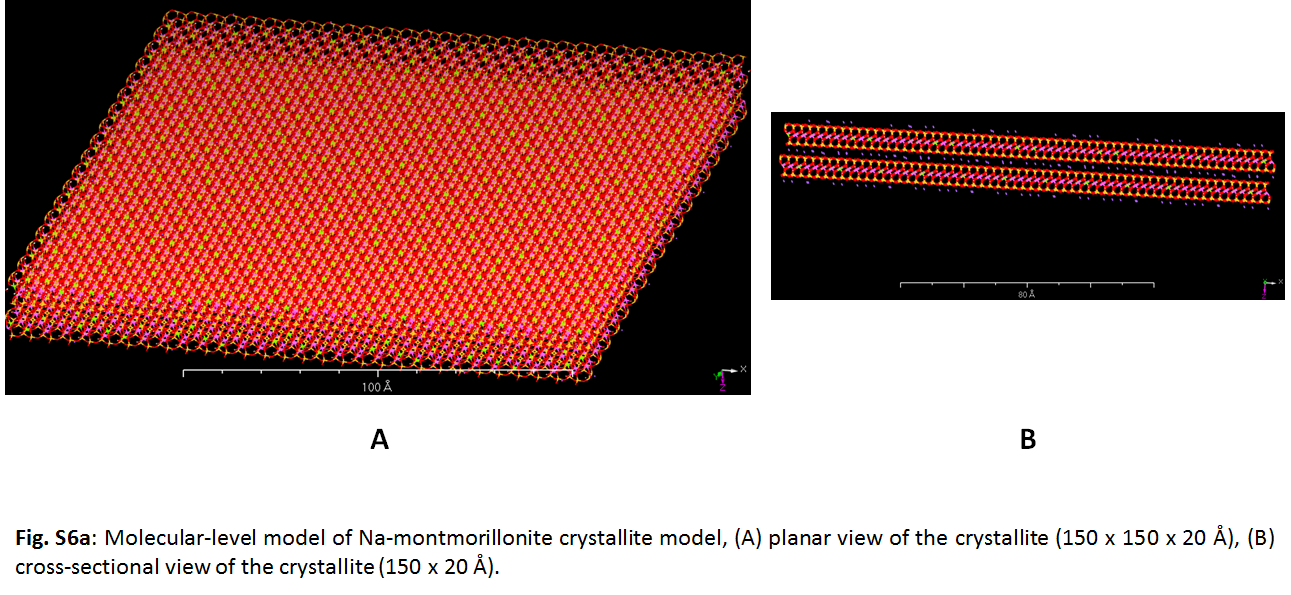


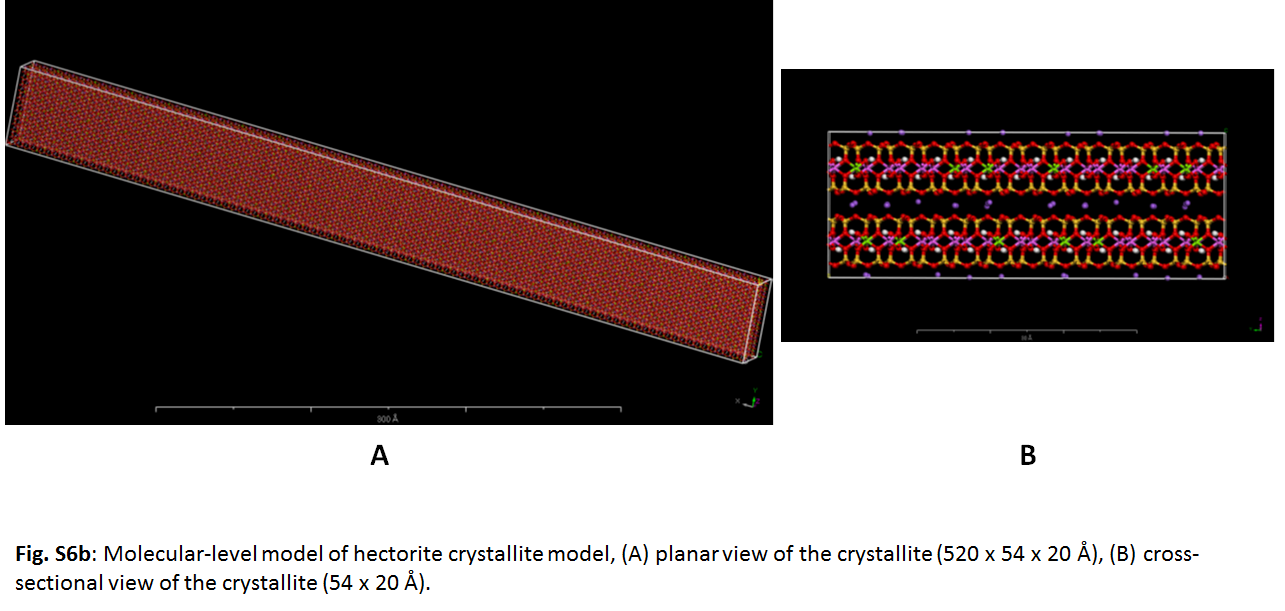


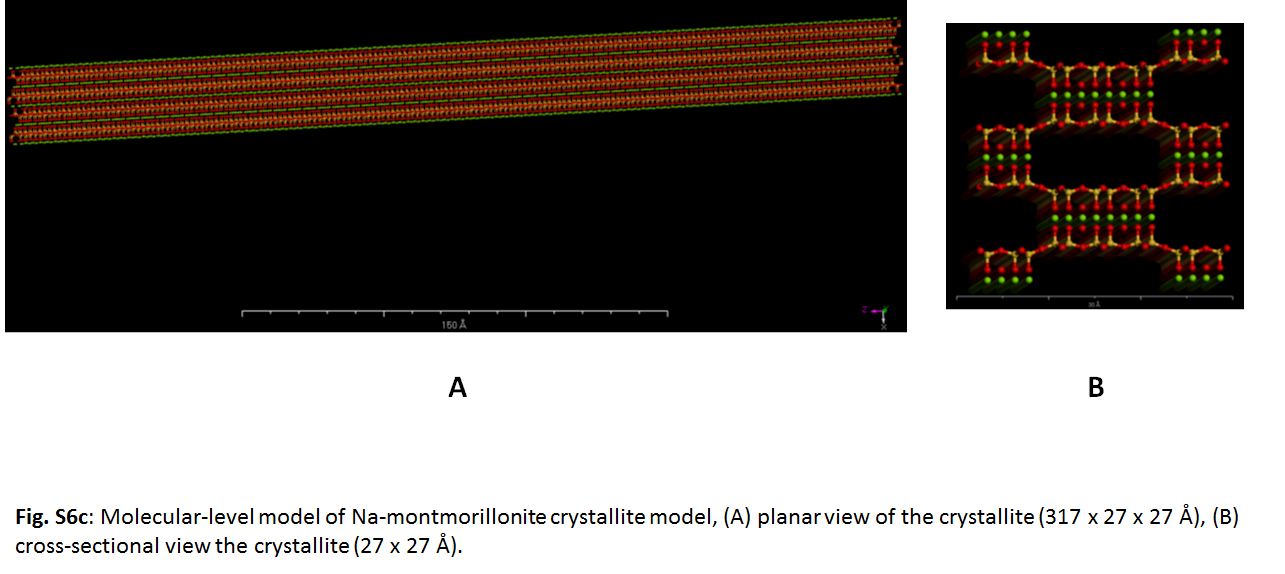


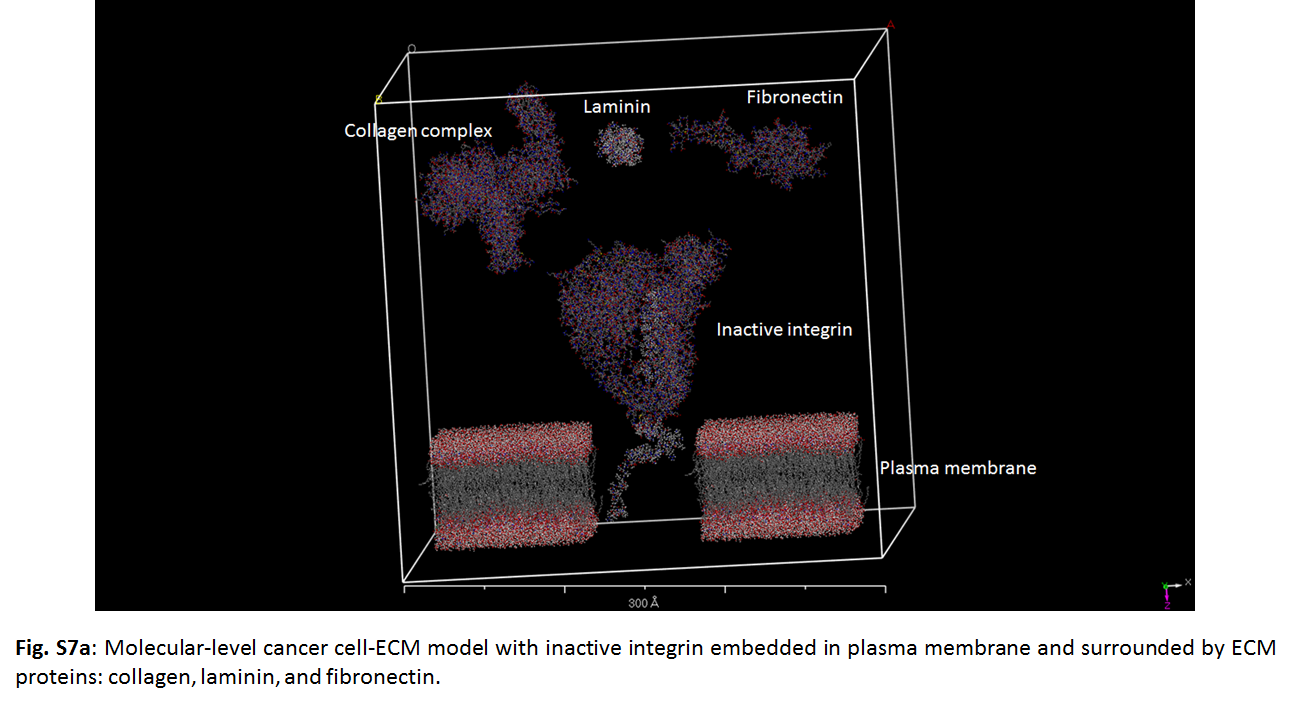


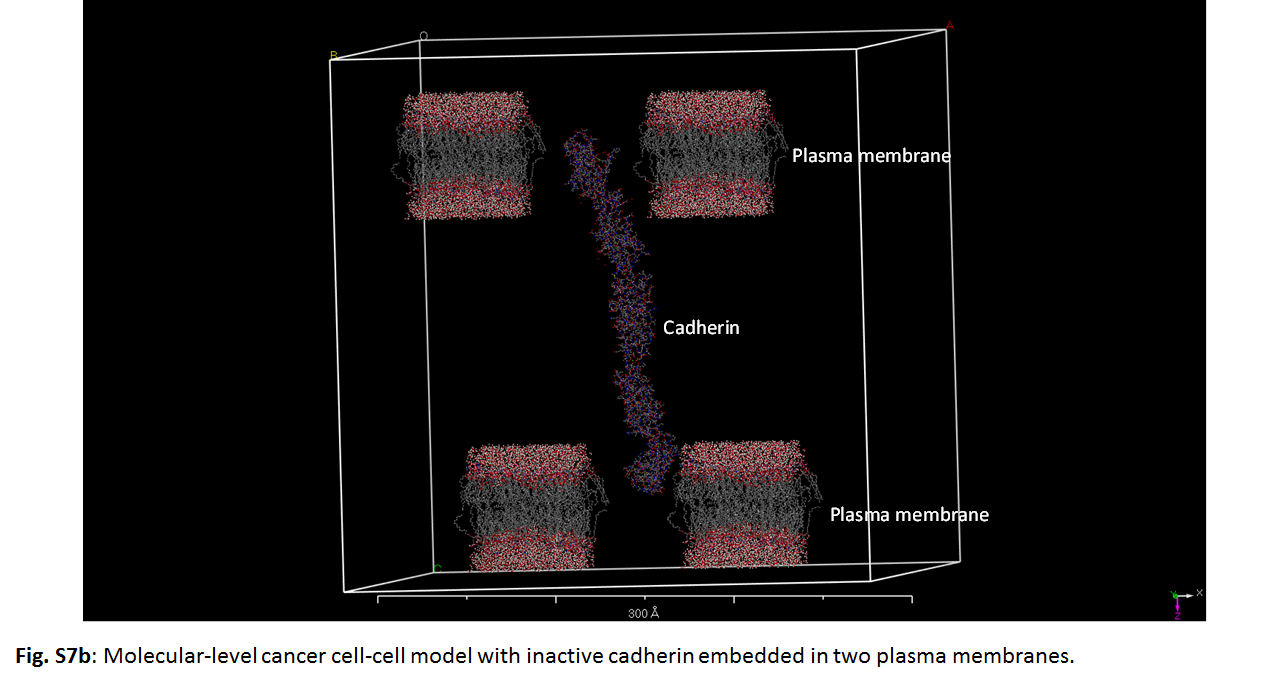


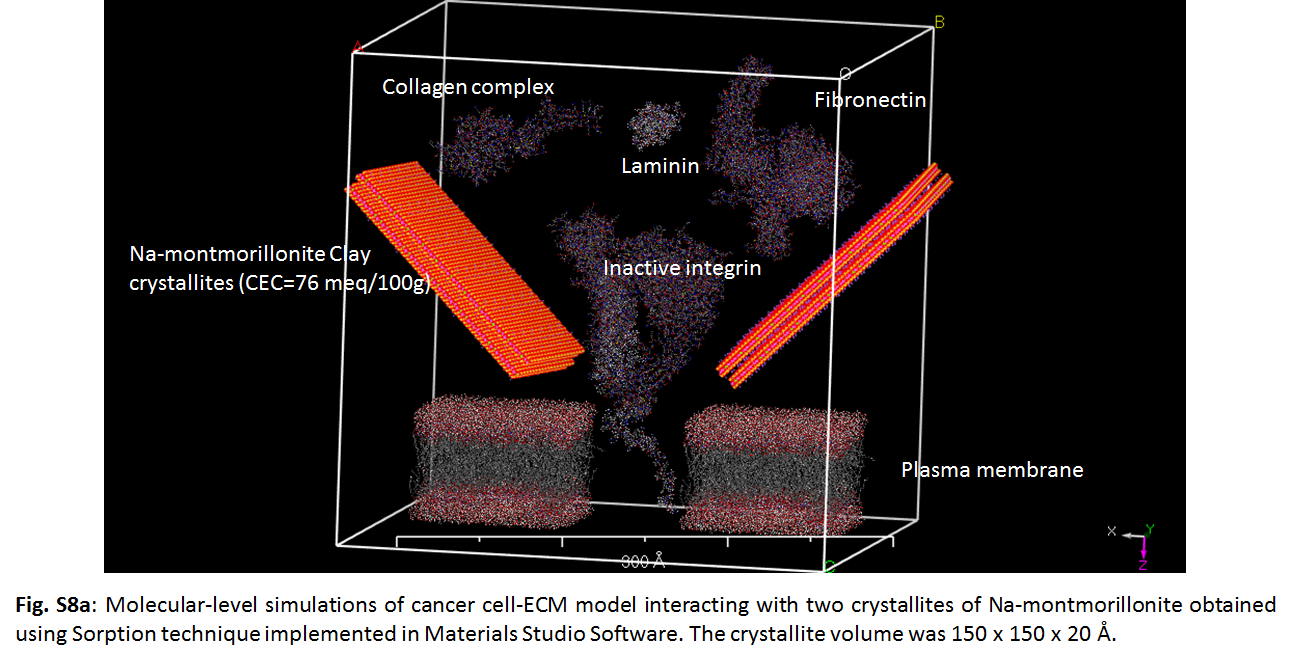


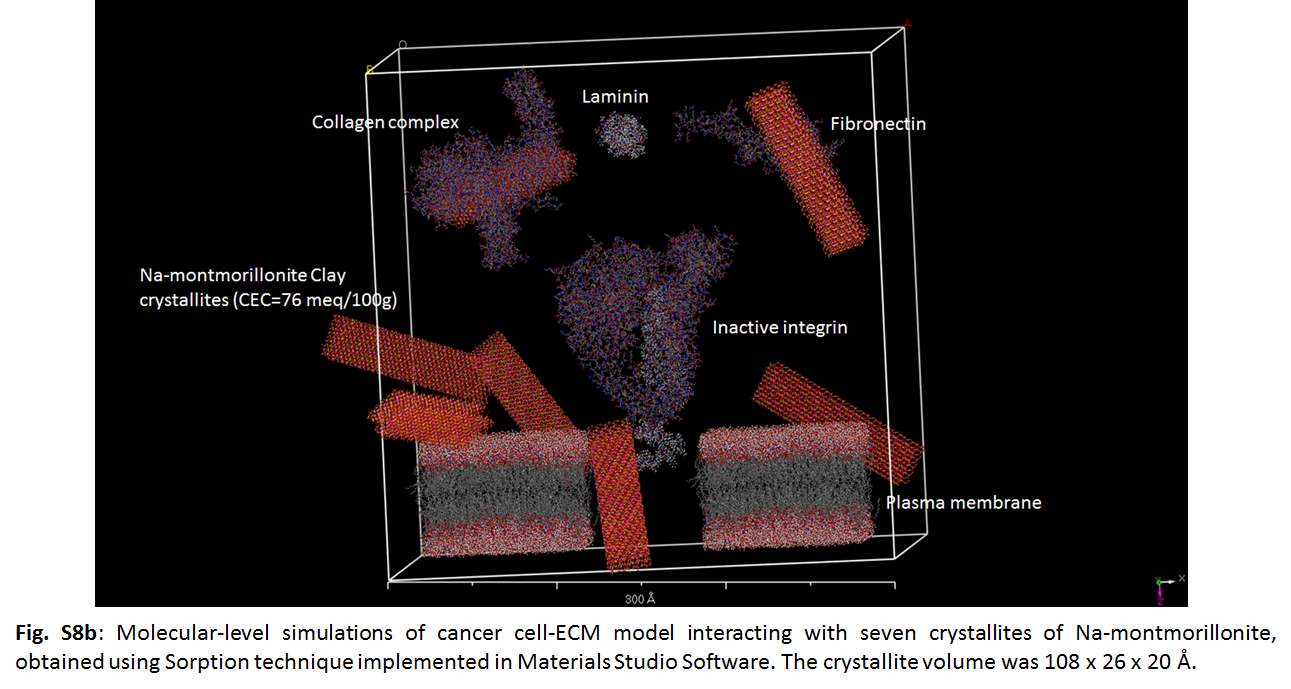


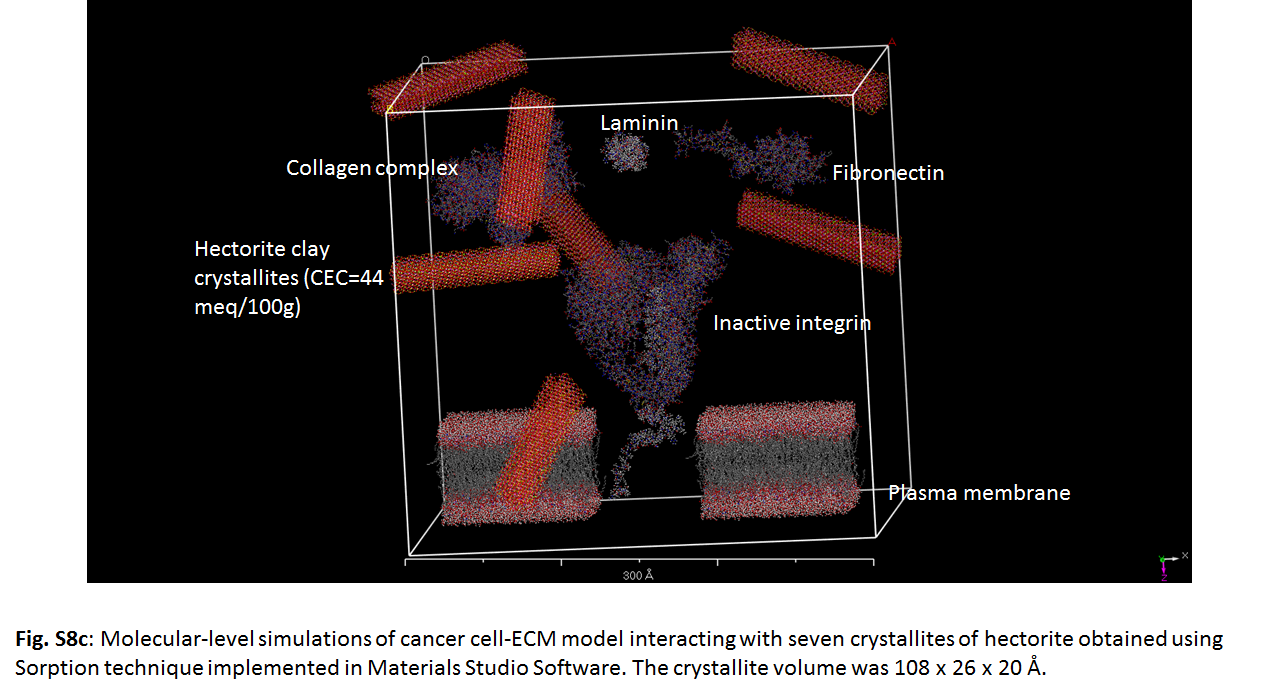


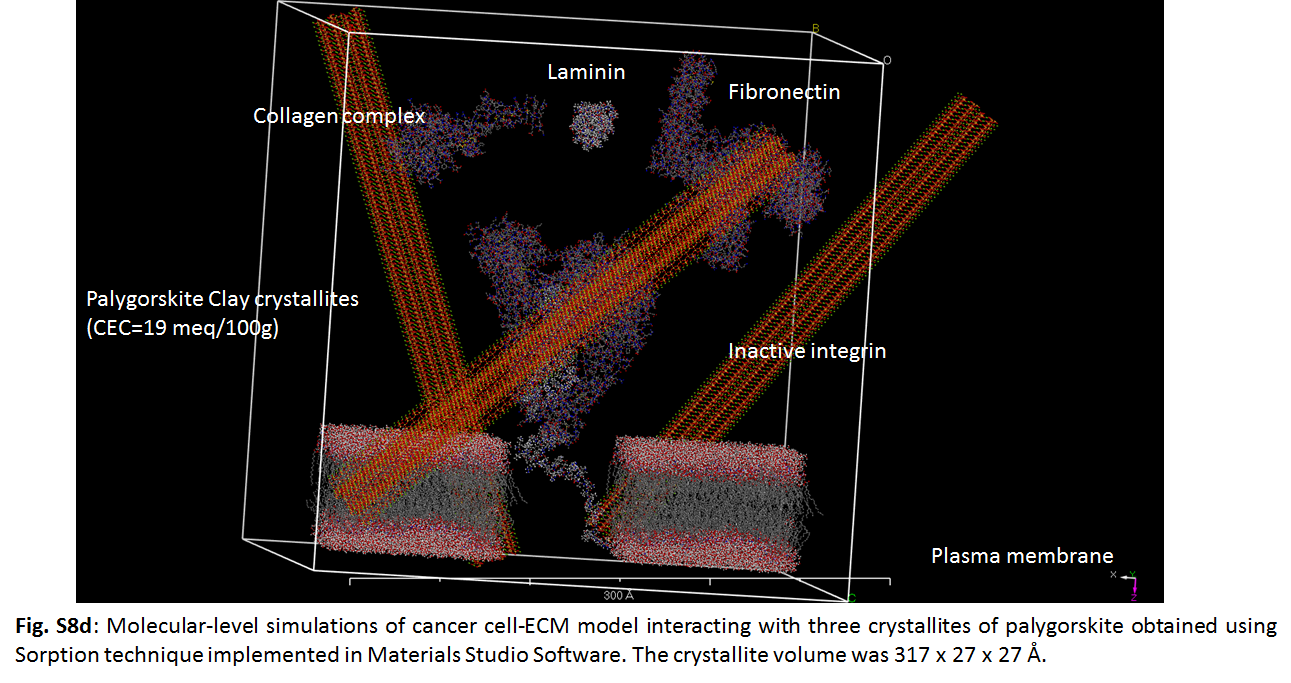


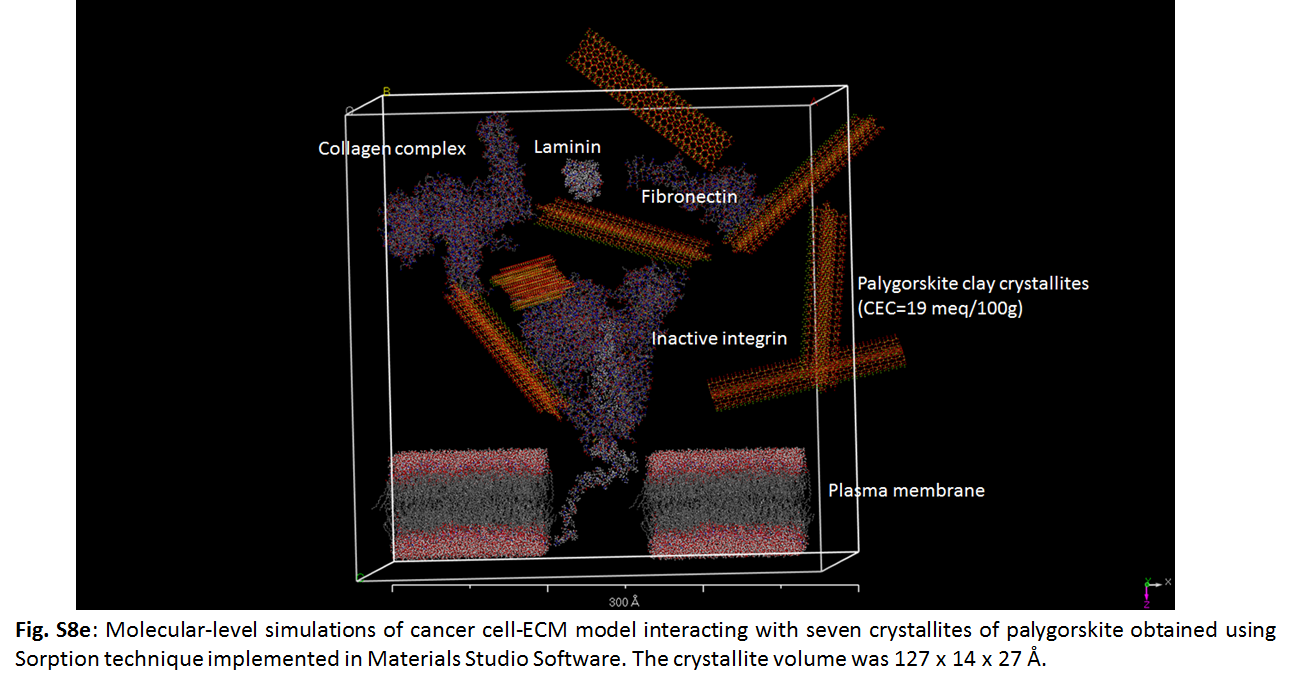


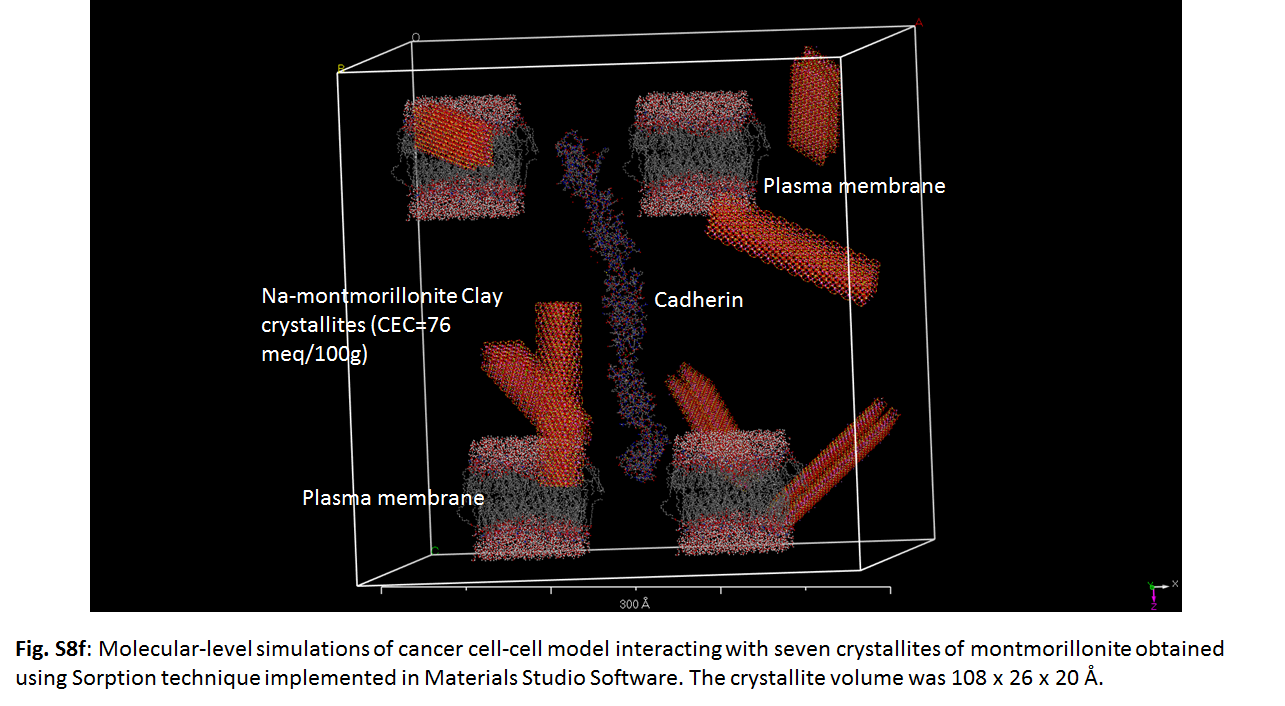


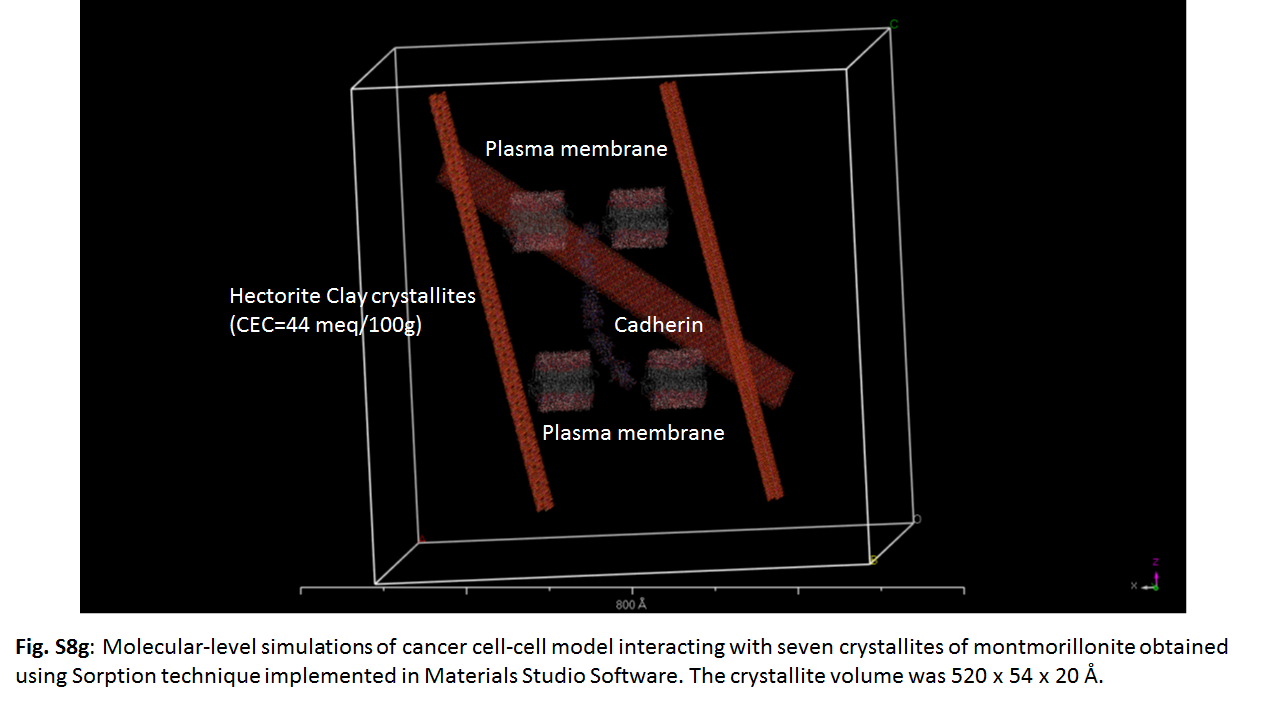

Supplement: Supplementary file 1 — Materials and Methods [file 41598_2019_42498_MOESM1_ESM.doc]
